# Risk of Hepatocellular Carcinoma With Tenofovir vs Entecavir Treatment for Chronic Hepatitis B Virus: A Reconstructed Individual Patient Data Meta-analysis

**DOI:** 10.1001/jamanetworkopen.2022.19407

**Published:** 2022-06-29

**Authors:** Darren Jun Hao Tan, Cheng Han Ng, Phoebe Wen Lin Tay, Nicholas Syn, Mark D. Muthiah, Wen Hui Lim, Ansel Shao Pin Tang, Kai En Lim, Grace En Hui Lim, Nobuharu Tamaki, Beom Kyung Kim, Margaret Li Peng Teng, James Fung, Rohit Loomba, Mindie H. Nguyen, Daniel Q. Huang

**Affiliations:** 1 Yong Loo Lin School of Medicine, National University of Singapore, Singapore; 2 Division of Gastroenterology and Hepatology, Department of Medicine, National University Hospital, Singapore; 3 National University Centre for Organ Transplantation, National University Health System, Singapore; 4 Lee Kong Chian School of Medicine, Nanyang Technological University, Sinagpore; 5 Department of Gastroenterology and Hepatology, Musashino Red Cross Hospital, Tokyo, Japan; 6 Department of Internal Medicine, Yonsei University College of Medicine, Seoul, South Korea; 7 Yonsei Liver Center, Severance Hospital, Yonsei University Health System, Seoul, South Korea; 8 Division of Liver Transplantation, Department of Surgery, Queen Mary Hospital, Hong Kong; 9 NAFLD (Nonalcoholic Fatty Liver Disease) Research Center, Division of Medicine, University of California, San Diego, La Jolla; 10 Division of Gastroenterology and Hepatology, Stanford University Medical Center, Palo Alto, California

## Abstract

**Importance:**

Conventional meta-analyses with aggregated study-level data have yielded conflicting results for the comparative effectiveness of tenofovir disoproxil fumarate vs entecavir in reducing hepatocellular carcinoma (HCC) risk among patients with chronic hepatitis B virus. Within-study heterogeneity, between-study heterogeneity, and the inability of conventional meta-analyses to capture time-to-event data were associated with these results.

**Objective:**

To perform a reconstructed individual patient data meta-analysis of high-quality propensity score–matched studies to provide robust estimates for comparative HCC risk between groups receiving tenofovir or entecavir.

**Data Sources:**

Medline and Embase databases were searched from inception to October 6, 2021.

**Study Selection:**

The initial search yielded 3435 articles. Fourteen studies that used propensity score matching to balance baseline characteristics were included in the final analysis.

**Data Extraction and Synthesis:**

The Preferred Reporting Items for Systematic Reviews and Meta-analyses guideline was followed. Individual patient data were reconstructed from Kaplan-Meier curves. Risk of HCC was evaluated using random-effects hazard ratios (HRs) via a shared-frailty model and a Cox proportional hazards model stratified by study group. Restricted mean survival time (RMST) analysis was conducted to account for varying estimated treatment effect across time.

**Main Outcomes and Measures:**

The comparative risk of HCC with tenofovir vs entecavir treatment.

**Results:**

From analysis of 14 studes with 24 269 patients (10 534 receiving tenofovir and 13 735 receiving entecavir; mean age, 49.86 [95% CI, 48.35-51.36] years; 65.05% [95% CI, 58.60%-71.00%] men), tenofovir was associated with decreased HCC incidence compared with entecavir (stratified Cox HR, 0.85 [95% CI, 0.76-0.94] at 5 years; *P* = .002). However, there was no significant difference in subanalysis of clinical cohort studies (stratified Cox HR, 0.92 [95% CI, 0.80-1.06] at 5 years; *P* = .24). Among administrative database studies, proportionality was violated, and HRs could not be obtained via Cox proporational hazards–based models. The mean time to HCC development in RMST analysis was 2.8 (95% CI, 1.8-3.7) weeks longer (*P* < .001) for tenofovir vs entecavir at 5 years. The RMST analyses for other subgroups revealed either insignificant or minimal differences (<3 weeks) in the mean time to HCC at 5 years.

**Conclusions and Relevance:**

In this meta-analysis, there was no clinically meaningful difference in the risk of HCC between patients who received entecavir and patients who received tenofovir. There was no difference between tenofovir and entecavir among clinical cohort studies, whereas the mean time to HCC development was less than 3 weeks longer for patients who received tenofovir vs those who received entecavir at year 5 among administrative database studies. The choice between tenofovir or entecavir should be decided based on patient convenience and tolerability.

## Key Points

**Question:**

What is the comparative risk of hepatocellular carcinoma (HCC) in patients with chronic hepatitis B virus infection receiving tenofovir vs entecavir?

**Findings:**

In this reconstructed individual patient data meta-analysis involving 24 269 patients (10 534 receiving tenofovir and 13 735 receiving entecavir) from 14 studies, tenofovir was associated with reduced HCC incidence. However, there were no differences among clinical cohort studies that likely provided better balancing of baseline characteristics between groups, whereas the mean time to HCC development was only 2.8 weeks longer in the tenofovir group than in the entecavir group at year 5 among administrative database studies.

**Meaning:**

These findings suggest that there is no clinically meaningful difference in the risk of HCC between entecavir and tenofovir and that the choice of treatment should be based on patient convenience and tolerability.

## Teaser

This meta-analysis reconstructs individual participant data from high-quality, propensity score–matched cohort studies and provides more accurate and precise estimates of hepatocellular carcinoma (HCC) risk among patients with chronic hepatitis B virus.

## Introduction

Chronic hepatitis B virus (CHBV) affects more than 250 million individuals worldwide and is the dominant risk factor for hepatocellular carcinoma (HCC).^1,2^ Nucleos(t)ide analogues reduce the risk of HCC in patients with CHBV by inhibiting viral replication and preventing fibrosis.^3,4,5^ Tenofovir and entecavir are highly potent nucleos(t)ide analogues with high genetic barriers to resistance and are both recommended by major society guidelines as first-line agents for the treatment of patients with CHB at higher risk for disease progression.^6,7,8,9,10,11^ However, the comparative effectiveness of tenofovir and entecavir in preventing HCC remains a matter of controversy and debate.^12^

Recently, several meta-analyses on the risk of HCC in patients with CHBV receiving tenofovir vs entecavir^13,14,15,16,17,18,19,20,21^ have reported conflicting results, with some favoring tenofovir and others reporting no significant difference in comparison with entecavir. Heterogeneity within studies, lack of appropriate subgroup analyses, and in particular differences between the 2 study groups with regard to follow-up times and differential background risks associated with selection bias that may not have been adequately adjusted for, especially in studies relying on administrative databases,^12^ contributed to these conflicting findings. In fact, study heterogeneity^21^ was resolved in 1 prior meta-analysis in subgroup analysis by study setting, with a significant difference in HCC risk observed in the subgroup analysis of administrative database studies but not in clinical cohort studies. However, all previous meta-analyses^14,15,21^ only relied on pooling study-level data, and time-to-event data were not captured for individual patients, limiting the precision of their estimates of HCC incidence due to lack of individual patient censoring.

Therefore, in the present study, we performed an individual patient data meta-analysis (IPDMA) using reconstructed individual participant data from high-quality, propensity score–matched cohort studies to provide more accurate and precise estimates of HCC risk to further clarify this controversy. An IPDMA is the criterion standard approach for pooled analysis of time-to-event data because it accounts for censoring and addresses between- and within-study heterogeneity.^22^ In addition and of particular importance, an IPDMA allows testing for violation of the proportional hazards assumption, which is a major requirement of the Cox proportional hazards model that prior conventional meta-analyses could not test for.^23^

## Methods

### Search Strategy

The synthesis of the literature was performed with reference to the Preferred Reporting Items for Systematic Review and Meta-Analyses (PRISMA) guideline for individual participant data systematic reviews.^24,25^ The search was performed on October 6, 2021, in the MEDLINE and Embase databases and started at their inception.^21^ Keywords and medical subject heading terms relating to *hepatocellular carcinoma*, *hepatitis B*, and *tenofovir* or *entecavir* were applied to the search strategy. The full search strategy can be found in the eMethods in the Supplement. All references were imported into Endnote, version X9 (Clarivate) for removal of duplicates. Manual screening of the references in the included articles was also conducted for a more comprehensive search.

### Study Eligibility and Selection Criteria

Four investigators (D.J.H.T., C.H.N., P.W.L.T., and W.H.L.) independently screened titles and abstracts and subsequently performed full-text reviews. Disputes were resolved through consensus from a senior author (D.Q.H.). The primary outcome was comparative HCC incidence among patients with CHBV receiving tenofovir disoproxil fumarate or entecavir. Only studies that directly compared outcomes in patients receiving tenofovir vs entecavir were considered for inclusion. In addition, we considered only high-quality propensity score–matched studies that included time-to-event data for the purpose of this IPDMA.^26,27^ Studies that matched patients by specified criteria, but did not calculate propensity scores, were also excluded from our analysis because there were insufficient matched variables to ensure an unbiased estimate of mean treatment effect.^28^ The process of propensity score matching is designed to minimize selection bias and to achieve balance in baseline characteristics between treatment groups. Existing statistical literature has demonstrated that the resultant effect estimates are empirically equivalent to those of a randomized clinical trial.^28,29,30^ Other study designs, including case-controlled or observational studies without matching, were excluded from the analysis. We reconstructed individual patient data via the model proposed by Guyot et al,^31^ using the original survival curves from the matched cohorts in the included studies. This method is the criterion standard approach in obtaining pooled estimates for survival analysis because it accounts for censoring of events and has been widely used in the extraction of data from published Kaplan-Meier curves.^27,32,33^

### Statistical Analysis

Analysis for the comparison of HCC incidence in patients receiving tenofovir vs those receiving entecavir was conducted using Cox proportional hazards models, and various approaches were used to address between-study heterogeneity.^26,34,35,36^ First, a stratified Cox model was used to obtain hazard ratios (HRs) of HCC incidence between patients receiving tenofovir vs entecavir, which models interstudy heterogeneity by assuming unique baseline hazards for each included study. In addition, we conducted analysis using a shared frailty model, which accounts for between-study heterogeneity by incorporating a random-effects model in which individual patients within each study are assumed to be similarly failure prone as other individuals belonging to that study, and frailties across studies are gamma distributed and affect the hazards function in a latent, multiplicative manner.^26,34,35,36^ All analyses were performed with follow-up truncated at 2 separate points (3 and 5 years) because the disparity between the numbers at risk between both groups increased after 3 years of follow-up. For the Cox proportional hazards–based models, we evaluated for proportionality (the assumption that the ratio of the risk of HCC between tenofovir and entecavir is constant over time) using the Grambsch-Therneau test for a nonzero slope with a visual representation of Schoenfeld residuals.^37^ Differences in HCC incidence between the 2 study groups were also assessed via nonparametric methods using restricted mean survival time (RMST) analysis.^38,39^ The RMST model provides a robust estimation of survival in the presence of proportionality violation and provides estimates for the difference in mean survival times (RMST difference) and the ratio of mean survival times between groups (RMST ratio). The RMST also accounts for varying treatment effect across time, and RMST differences and ratios can be calculated at specific cutoff points. As opposed to an HR, for which a value of less than 1.00 is associated with a better outcome, a larger RMST value represents longer event-free survival time and equates to improved clinical outcomes.

Subgroup analyses were conducted for the following groups: studies using administrative databases (including national electronic medical records or national insurance claims databases) and those from clinical cohort studies as well as single-center vs multicenter studies. Additional sensitivity analyses were conducted for studies involving only patients with cirrhosis and treatment-naive patients who did not receive previous nucleos(t)ide analogues before initiation of tenofovir or entecavir therapy. Statistical significance was defined as 2-tailed *P* < .05. The baseline characteristics of included patients were analyzed, both before and after propensity score matching, including age, sex, body mass index, presence of type 2 diabetes, presence of cirrhosis and decompensated cirrhosis, and HBV DNA titer and hepatitis Be antigen (HBeAg) status. Binary variables were analyzed using a meta-analysis of proportions with a generalized linear mixed model with Clopper-Pearson intervals, whereas continuous variables were analyzed using inverse weightage pooling in a DerSimonian-Laird random effects model.^40,41,42^ All statistical analyses were conducted using Rstudio, version 4.1.1 (R Project for Statistical Computing), or STATA, version 16.1 (StataCorp LLC), where appropriate.

We evaluated the quality of the included articles using the Newcastle Ottawa Scale. The Newcastle Ottawa Scale appraisal tool evaluates studies based on several parameters, including appropriateness of the sample frame, sampling method, ascertainment of exposure, demonstration that the outcome of interest was not present at start of study, comparability of cohorts, methods for assessment of outcomes, duration of follow-up, and adequacy of follow-up.^43^ Funnel plots were constructed for analyses involving more than 10 studies. Publication bias was assessed via visual inspection of funnel plots for asymmetrical distribution of the data points across the vertical treatment effect axis.^44^

## Results

### Summary of Included Articles

**Figure 1.**
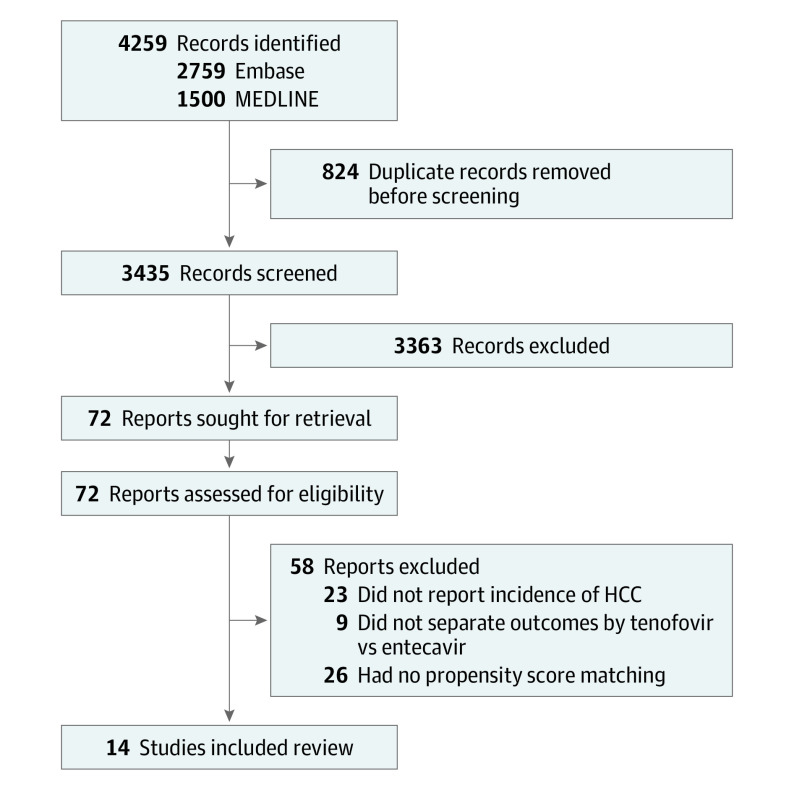
PRISMA Flowchart of Included Articles HCC indicates hepatocellular carcinoma.

The initial search yielded 3435 articles after removal of duplicates (Figure 1). After study title and abstract review, we excluded 3363 articles and retrieved 72 for full text review. Finally, 14 articles^45,46,47,48,49,50,51,52,53,54,55,56,57,58^ met the study inclusion criteria and were included in the study analysis. By geographic distribution, 8 studies were conducted in South Korea,^45,46,47,48,49,50,51,52^ 3 in Taiwan,^53,54,55^ 1 in mainland China,^56^ 1 in the US,^57^ and 1 with centers from both the US and Asia.^58^ A total of 11 studies^45,46,47,48,49,50,51,52,54,55,58^ (13 626 patients) used data from clinical cohorts, whereas 4 studies^45,53,56,57^ (32 489 patients) analyzed data from administrative databases. The study by Choi et al^45^ reported data from both a hospital cohort and a national insurance claims database from South Korea. Each of these 2 cohorts was included in subanalysis for clinical cohorts and administrative databases as applicable, whereas only the hospital-based cohort (n = 869) was included in the overall result of our pooled estimates, because the cohort from the national insurance claims database (n = 10 923) may have overlapped with several other clinical cohorts from South Korea in the same period.^45,46,47,48,49,50,51,52,59^ This resulted in a lower number of patients in the overall analysis compared with the subgroup analysis for administrative cohorts. eTable 1 in the Supplement summarizes the key characteristics and quality assessment for the included articles, whereas eFigure 1 in the Supplement shows the original and reconstructed survival curves from the included articles. All included studies were of high quality, with a Newcastle Ottawa Scale score of 8 or higher. No publication bias was detected from visual assessment of the funnel plots (eFigure 2 in the Supplement).

### Baseline Characteristics

The baseline characteristics of the patients who received tenofovir and the patients who received entecavir before and after propensity score matching are provided in Table 1 (mean age, 49.86 [95% CI, 48.35-51.36] years; 65.05% [95% CI, 58.60%-71.00%] men and 34.95% [95% CI, 28.90%-41.40%] women). Before propensity score matching, patients receiving entecavir were slightly older than those receiving tenofovir (51.48 [95% CI, 49.41-53.55] vs 48.90 [95% CI, 46.75-51.04] years; *P* = .09) and a greater proportion had type 2 diabetes (13.20% [95% CI, 10.39%-16.63%] vs 9.70% [95% CI, 7.62%-12.27%]; *P* = .07), although neither of these comparative analyses reached conventional level of statistical significance. After propensity score matching, there was no statistically significant difference in the included baseline characteristics between patients receiving tenofovir vs those receiving entecavir (Table 1). The median follow-up among the tenofovir vs entecavir groups was 3.46 (IQR, 1.95-4.80) vs 4.01 (IQR, 2.17-5.00) years.

**Table 1.** Summary of Baseline Characteristics Comparing Patients Receiving Tenofovir vs Entecavir Before and After Propensity Score Matching

Characteristic	No. of studies	Tenofovir cohort		Entecavir cohort		*P* value^a^
		Total No. of patients	Value (95% CI)	Total No. of patients	Value (95% CI)	
Before propensity score matching						
Age, mean, y	12	11 415	48.90 (46.75-51.04)	47 813	51.48 (49.41-53.55)	.09
Sex, %						
Women	12	11 415	34.87 (31.82-38.05)	47 813	34.75 (32.08-37.52)	.95
Men	12	11 415	65.13 (61.94-68.18)	47 813	65.25 (62.48-67.92)	
Mean BMI	6	6087	23.93 (23.13-24.74)	14 851	23.94 (23.13-24.75)	.99
Type 2 diabetes, %	12	11 415	9.70 (7.62-12.27)	47 813	13.20 (10.39-16.63)	.07
Cirrhosis, %	12	11 415	63.92 (27.78-89.08)	47 813	51.52 (18.79-82.99)	.64
Decompensated cirrhosis, %	3	7094	10.82 (7.11-16.12)	3274	14.11 (7.82-24.13)	.46
HBeAg postive, %	12	11 415	46.76 (37.32-56.44)	47 813	41.68 (31.44-52.69)	.49
HBV DNA, mean log IU/mL	11	11 199	5.56 (4.95-6.17)	47 135	5.85 (5.30-6.40)	.49
ALT level, mean, U/L	7	5567	121.23 (90.74-151.72)	11 266	134.77 (108.45-161.10)	.51
After propensity score matching						
Age, mean, y	14	10 420	49.70 (47.64-51.74)	13 969	50.01 (47.73-52.29)	.84
Sex, %						
Women	14	10 420	33.21 (26.46-40.73)	13 969	32.42 (24.83-41.05)	.89
Men	14	10 420	64.88 (55.99-72.85)	13 969	65.22 (55.66-73.69)	.91
Mean BMI	7	6657	24.49 (23.15-25.82)	9921	24.56 (23.29-25.82)	.94
Type 2 diabetes, %	13	10 542	10.80 (8.34-13.87)	13 808	10.72 (8.31-13.72)	.97
Cirrhosis, %	14	10 420	50.68 (20.57-80.31)	13 969	55.19 (20.69-85.33)	.86
Decompensated cirrhosis, %	4	4885	10.41 (7.17-14.87)	3233	8.66 (5.47-13.46)	.53
HBeAg positive, %	13	10 542	43.51 (34.85-52.58)	13 808	42.48 (32.58-53.03)	.88
HBV DNA, mean log IU/mL	10	7639	5.90 (5.50-6.30)	7702	5.90 (5.49-6.31)	>.99
ALT level, mean U/L	7	4216)	125.77 (105.32-146.22)	4666	124.89 (106.45-143.33)	.95

Abbreviations: ALT, alanine transaminase; BMI, body mass index (calculated as weight in kilograms divided by height in meters squared); HBeAg, hepatitis Be antigen; HBV, hepatitis B virus.

SI conversion factor: To convert ALT to μkat/L, multiply by 0.0167.

^a^ Comparison of baseline characteristics between patients receiving tenofovir and patients receiving entecavir.

### Analysis of the Overall Cohort

**Figure 2.**
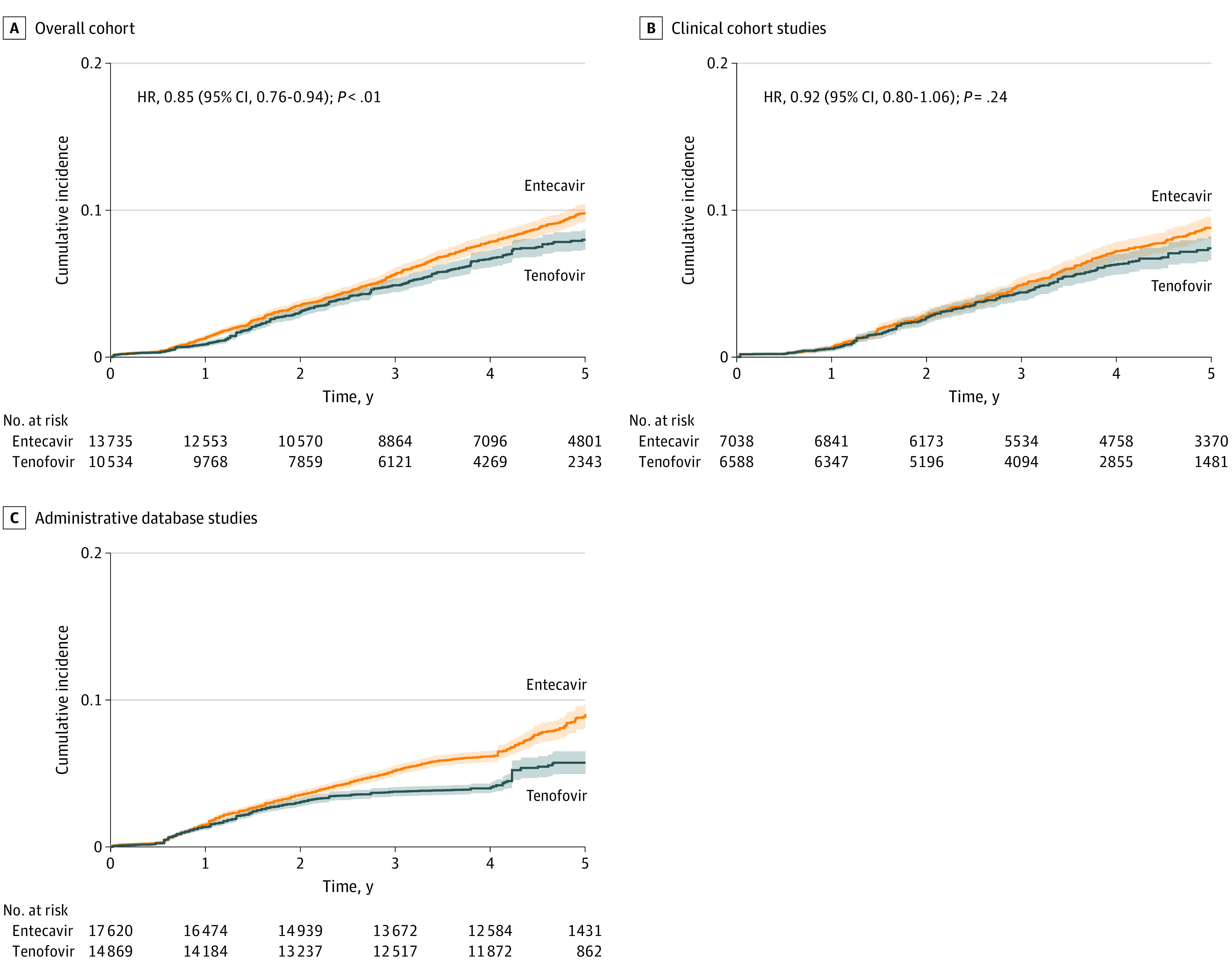
Cumulative Incidence of Hepatocellular Carcinoma in Patients Receiving Tenofovir vs Entecavir HR indicates hazard ratio. Shaded areas indicate 95% CIs.

A total of 24 269 patients from 14 studies (10 534 receiving tenofovir and 13 735 receiving entecavir) were evaluated for HCC incidence in the overall analysis (Figure 2A). The 1-year incidence of HCC in the tenofovir group was 3.08% (95% CI, 2.74%-3.45%); 3-year incidence, 6.71% (95% CI, 6.16%-7.31%); and 5-year incidence, 8.26% (95% CI, 7.57%-9.01%). In the entecavir group, 1-year incidence of HCC was 3.51% (95% CI, 3.20%-3.86%); 3-year incidence, 7.85% (95% CI, 7.34%-8.38%); and 5-year incidence, 10.87% (95% CI, 10.19%-11.61%). In the stratified Cox model, tenofovir was associated with decreased HCC incidence vs entecavir when follow-up was censored at 3 years (HR, 0.87 [95% CI, 0.77-0.99]; *P* = .03) and after 5 years (HR, 0.85 [95% CI, 0.76-0.94]; *P* = .002). Analysis via the shared frailty model to help account for between-study heterogeneity yielded similar estimates (Table 2).

**Table 2.** Summary of Analysis of HCC Incidence in Patients Receiving Tenofovir vs Entecavir in Overall and Subgroup Analyses

Variable	3-y Follow-up		5-y Follow-up	
	HR (95% CI)^a^	*P* value	HR (95% CI)^a^	*P* value
Overall analysis				
Random-effects HR (shared frailty)	0.87 (0.77-0.98)	.02	0.84 (0.76-0.94)	.001
Stratified Cox HR	0.87 (0.77-0.99)	.03	0.85 (0.76-0.94)	.002
Patients with cirrhosis only				
Random-effects HR (shared frailty)	0.46 (0.37-0.57)	<.001	0.55 (0.46-0.66)	<.001
Stratified Cox HR	0.63 (0.52-0.76)	<.001	0.67 (0.56-0.79)	<.001
Clinical cohorts				
Random-effects HR (shared frailty)	0.95 (0.80-1.12)	.53	0.92 (0.80-1.06)	.26
Stratified Cox HR	0.96 (0.81-1.14)	.62	0.92 (0.80-1.06)	.24
Administrative database, proportionality violated				
Single-center studies				
Random-effects HR (shared frailty)	0.85 (0.67-1.07)	.16	0.77 (0.64-0.94)	.01
Stratified Cox HR	0.87 (0.69-1.10)	.24	0.77 (0.63-0.94)	.01
Multicenter studies				
Random-effects HR (shared frailty)	0.89 (0.77-1.03)	.13	0.83 (0.73-0.94)	.003
Stratified Cox HR	0.89 (0.77-1.03)	.13	0.83 (0.73-0.94)	.004
Treatment naive				
Random-effects HR (shared frailty)	0.79 (0.68-0.93)	.004	0.80 (0.70-0.92)	.001
Stratified Cox HR	0.80 (0.68-0.93)	.004	0.80 (0.70-0.91)	.001

Abbreviations: HCC, hepatocellular carcinoma; HR, hazard ratio.

^a^ Calculated with entecavir as the reference group.

The RMST analysis was conducted to evaluate differences in HCC incidence between the patients who received tenofovir and the patients who received entecavir in the overall cohort over time. There was a statistically significant longer mean time to HCC occurrence from 2 years of follow-up onward in patients receiving tenofovir compared with entecavir, although the difference was modest (RMST difference at 2 years, 0.006 [95% CI, 0.001-0.010] years [*P* = .02]; RMST difference at 5 years, 0.033 [95% CI, 0.011-0.055] years; [*P* < .001]) (eFigure 2A in the Supplement).

### Subgroup and Sensitivity Analyses

#### Clinical Cohort Studies

A total of 11 studies^45,46,47,48,49,50,51,52,54,55,58^ (13 626 patients; 6588 receiving tenofovir and 7038 receiving entecavir) used data from hospital-based clinical cohorts. There was no significant difference in HCC incidence (Figure 2B) between tenofovir vs entecavir when follow-up was censored at 3 years (stratified Cox HR, 0.96 [95% CI, 0.81-1.14]; *P* = .62) or 5 years (stratified Cox HR, 0.92 [95% CI, 0.80-1.06]; *P* = .24), with consistent results in analysis from the shared frailty model and RMST analysis (Table 2 and Table 3).

**Table 3.** RMST Analysis of HCC Incidence in Patients Treated With Tenofovir vs Entecavir Stratified by Clinical Cohort or Administrative Database Studies

Follow-up year	RMST (95% CI), y		RMST difference (95% CI), y	*P* value	RMST ratio (95% CI)	*P* value
	Tenofovir	Entecavir				
Clinical cohort						
1	0.997 (0.996 to 0.998)	0.997 (0.996 to 0.998)	0.000 (−0.002 to 0.002)	.99	1.000 (0.998 to 1.002)	.99
2	1.981 (1.978 to 1.985)	1.980 (1.976 to 1.983)	0.002 (−0.003 to 0.006)	.53	1.001 (0.999 to 1.003)	.53
3	2.945 (2.938 to 2.953)	2.942 (2.935 to 2.949)	0.003 (−0.007 to 0.014)	.53	1.001 (0.998 to 1.005)	.53
4	3.892 (3.879 to 3.904)	3.882 (3.870 to 3.894)	0.009 (−0.008 to 0.027)	.29	1.002 (0.998 to 1.007)	.29
5	4.823 (4.804 to 4.842)	4.803 (4.785 to 4.821)	0.020 (−0.006 to 0.046)	.13	1.004 (0.999 to 1.010)	.13
Administrative studies						
1	0.995 (0.994 to 0.996)	0.995 (0.994 to 0.996)	0.000 (−0.001 to 0.001)	.99	1.000 (0.999 to 1.001)	.99
2	1.972 (1.969 to 1.975)	1.969 (1.966 to 1.972)	0.003 (−0.001 to 0.007)	.14	1.002 (0.999 to 1.004)	.14
3	2.937 (2.932 to 2.943)	2.926 (2.920 to 2.931)	0.011 (0.003 to 0.019)	.005	1.004 (1.001 to 1.007)	.005
4	3.899 (3.890 to 3.907)	3.868 (3.860 to 3.877)	0.030 (0.018 to 0.042)	<.001	1.008 (1.005 to 1.011)	<.001
5	4.864 (4.833 to 4.859)	4.793 (4.781 to 4.806)	0.053 (0.035 to 0.071)	<.001	1.011 (1.007 to 1.015)	<.001

Abbreviations: HCC, hepatocellular carcinoma; RMST, restricted mean survival time.

#### Administrative Database Studies

A total of 32 489 patients (14 869 receiving tenofovir and 17 620 receiving entecavir) from 4 studies^45,53,56,57^ used data from administrative databases (Figure 2C). The proportionality assumption was not met; therefore, the shared frailty and Cox proportional hazards regression model analyses were not performed. In the RMST analysis, tenofovir was associated with longer mean time to HCC compared with entecavir from the 3-year follow-up onward, although the difference was modest (RMST difference at 3 years, 0.011 [95% CI, 0.003-0.019] years [*P* = .005]; RMST difference at 5 years, 0.053 [95% CI, 0.035-0.071] years [*P* < .001]) (Table 3).

#### Cirrhosis

A total of 9 studies^45,46,47,49,50,51,52,53,54,55^ (6083 patients; 2677 receiving tenofovir and 3406 receiving entecavir) provided data for HCC incidence among patients with cirrhosis (eFigure 3 in the Supplement). The 1-year incidence of HCC in the tenofovir was 6.25% (95% CI, 5.36%-7.28%); 3-year incidence, 12.22% (95% CI, 10.85%-13.75%); and 5-year incidence, 13.93% (95% CI, 12.37%-15.67%). In the entecavir group, 1-year incidence of HCC was 7.05% (95% CI, 6.21%-7.99%); 3-year incidence, 14.28% (95% CI, 13.06%-15.61%); and 5-year incidence, 17.47% (95% CI, 16.01%-19.04%). Proportionality was not violated in the sensitivity analysis for patients with cirrhosis. Tenofovir was associated with lower risk of HCC compared with entecavir when follow-up was censored at 3 years (stratified Cox HR, 0.63 [95% CI, 0.52-0.76]; *P* < .001) and 5 years (stratified Cox HR, 0.67 [95% CI, 0.56-0.79]; *P* < .001). Analysis using a shared frailty model also revealed lower HCC risk in patients receiving tenofovir (Table 2). However, RMST analysis revealed no statistically significant difference in the mean time to HCC between the 2 study groups, even at 5 years of follow-up (RMST difference at 5 years, 0.050 [95% CI, −0.010 to 0.109] years; *P* = .10) (eTable 2A in the Supplement).

#### Single-Center Studies

A total of 7908 patients from 7 single-center studies^45,47,48,49,51,52,54^ were included in this subgroup analysis (3729 receiving tenofovir and 4179 receiving entecavir). There was similar risk of HCC between tenofovir and entecavir when follow-up was censored at 3 years (stratified Cox HR, 0.87 [95% CI, 0.69-1.10]; *P* = .24), but lower risk of HCC in the tenofovir group when follow-up was censored at 5 years (stratified Cox HR, 0.77 [95% CI, 0.63-0.94]; *P* = .01), with similar findings in the shared frailty model (eFigure 4A in the Supplement and Table 2). Likewise, RMST analysis found a statistically significant longer mean time to HCC occurrence in the tenofovir group only at 5 years, although the effect size was also modest (RMST difference at 5 years, 0.042 [95% CI, 0.006-0.077] years; *P* = .02) (eTable 2B in the Supplement).

#### Multicenter Studies

This subgroup analysis included a total of 7 studies^46,50,53,55,56,57,58^ and 16 361 patients (6805 receiving tenofovir and 9556 receiving entecavir). Tenofovir was associated with similar HCC incidence compared with entecavir when follow-up was censored at 3 years (stratified Cox HR, 0.89 [95% CI, 0.77-1.03]; *P* = .13). However, patients receiving tenofovir had a lower HCC risk when follow-up was censored at 5 years (stratified Cox HR, 0.83 [95% CI, 0.73-0.94]; *P* = .004) (eFigure 4B in the Supplement and Table 2). Similar findings were observed at both time points in analysis via a shared frailty model (Table 2). However, in RMST analysis, there was no statistically significant difference in the mean time to HCC occurrence in the 2 study groups to 5 years of follow-up (RMST difference at 5 years, 0.028 [95% CI, 0.000-0.056] years; *P* = .05) (eTable 2B in the Supplement).

#### Studies Involving Only Treatment-Naive Patients

This analysis included 11 studies^45,46,47,48,49,50,51,52,54,56,58^ of only treatment-naive patients before initiation of tenofovir or entecavir therapy (14 936 patients; 7243 receiving tenofovir and 7693 receiving entecavir). Patients receiving tenofovir had significantly lower incidence (eFigure 5 in the Supplement) and risk of HCC compared with entecavir at 3 years (stratified Cox HR, 0.80 [95% CI, 0.68-0.93]; *P* = .004) and at 5 years (stratified Cox HR, 0.80 [95% CI, 0.70-0.91]; *P* = .001), with similar findings in analysis using the shared frailty model (Table 2). The RMST analysis also found longer mean time to HCC occurrence in patients who received tenofovir starting at 2 years of follow-up, but the difference was only 0.049 (95% CI, 0.022-0.075) years, even at 5 years of follow-up (*P* <. 001) (eTable 2C in the Supplement).

## Discussion

### Main Findings

In this reconstructed IPDMA of 24 269 patients with CHBV (10 534 receiving tenofovir and 13 735 receiving entecavir), there was no clinically meaningful difference in the risk of HCC between those receiving tenofovir and those receiving entecavir. Although there was a longer mean time to HCC development with tenofovir vs entecavir in the overall analysis, the difference was modest (0.006 years or 0.30 weeks at 2 years, and 0.033 years or 1.72 weeks at 5 years) and unlikely to be clinically significant. These data suggest that factors such as cost, drug availability, and comorbidities should be the main consideration when deciding between tenofovir or entecavir. In subgroup analysis of administrative database studies, the mean time to HCC among patients receiving tenofovir was modestly longer compared with patients receiving entecavir (0.011 years or 0.57 weeks at year 3, and 0.053 years or 2.76 weeks at year 5); and because the proportionality assumption was not met, the shared frailty and Cox proportional hazards regression models were not performed for this subgroup. In subgroup analysis of clinical cohort studies, which may have had more detailed clinical data to adjust for confounders, there was no significant difference between the 2 groups in stratified Cox analysis. Additional analysis via a shared frailty model to account for between-study heterogeneity and RMST analysis to account for varying treatment effect across time revealed similar findings between tenofovir and entecavir in clinical cohort studies. In the absence of high-quality randomized clinical trials, the present analysis provides the highest level of evidence available to date evaluating the comparative risk of HCC between tenofovir and entecavir.

The lower incidence of HCC among patients treated with tenofovir vs entecavir persisted in subgroup analysis for patients with cirrhosis and for patients who were treatment naive before tenofovir or entecavir. However, RMST analysis for both subgroups determined that the mean time to HCC was only modestly longer (0.050 years or 2.6 weeks at 5 years) among patients receiving tenofovir vs entecavir. The subgroup analyses for multicenter and single-center studies showed no significant difference in HCC risk between the 2 study groups at 3-year follow-up, however, a lower risk was observed at 5-year follow-up in patients receiving tenofovir. This may indicate that time is required for the beneficial effect of tenofovir on hepatocarcinogenesis. On the other hand, the difference between tenofovir and entecavir may be related to imbalance between the 2 groups in the number of patients remaining at risk after 3 years, given the known differences in follow-up duration between the 2 study groups. Indeed, results from our RMST analyses suggest that this may be the case because we found no significant difference in the mean time to HCC development between the 2 groups for multicenter studies and only marginally longer time to HCC development (2.18 weeks) with tenofovir at 5-year follow up.

The potential mechanism for the lower HCC risk in patients receiving tenofovir vs entecavir, if any, is unclear.^60,61^ A recent meta-analysis^62^ reported variable early comparative results with regard to viral suppression rates between patients receiving tenofovir vs entecavir with odds ratios of 1.12 (95% CI, 0.89-1.41) at 12 weeks, 1.33 (95% CI, 1.11-1.61) at 24 weeks, and 1.62 (95% CI, 1.16-2.25) at 48 weeks and nonsignificant results at 72 and 96 weeks (odds ratios, 1.43 [95% CI, 0.78-2.62] and 1.56 [95% CI, 0.87-2.81], respectively). These inconsistencies are likely owing to patient selection bias in observational studies and the high level of heterogeneity observed in these analyses with data derived from both randomized clinical trials and observational studies. In 1 study comparing patients receiving tenofovir and entecavir with HBV DNA levels greater than 1 million IU/mL, tenofovir was associated with a higher likelihood of viral suppression among patients with positive findings for HBeAg but not those with findings negative for HBeAg, although this study only included 20 patients positive for HBeAg receiving tenofovir and 135 receiving entecavir, thus limiting its conclusions.^62^ With regard to viral resistance, entecavir is associated with a low risk of resistance (1.2% after 5 years),^63^ whereas no resistance was observed after 8 years of tenofovir.^64^

### Context of Current Literature

Multiple meta-analyses^14,15,21^ have reported conflicting results on this topic. However, previously published meta-analyses did not account for censoring of patients during follow-up between the tenofovir and entecavir cohorts, which may have resulted in inaccurate estimations of HCC incidence. In addition, previous meta-analyses did not limit their inclusion criteria to propensity score–matched cohorts, which is an additional source of within-study heterogeneity due to the lack of controlling for baseline characteristics. Our study addresses many of the previous methodological issues with study level data meta-analyses on this topic by pooling time-to-event data from individual patient level data and by including only high-quality propensity score–matched studies. We also conducted several additional analyses using the shared frailty and stratified Cox models to account for between-study heterogeneity and RMST analysis to account for varying treatment effect across time, which is an important consideration for this topic because most tenofovir cohorts had shorter follow-up than the entecavir cohorts. In addition, when the proportional hazards assumption was found to be violated, we used nonparametric RMST analysis to evaluate differences in HCC incidence instead. The RMST model has been found to yield more robust estimates in such situations, compared with alternatives such as Cox proportional hazards regression analysis, which can overestimate or underestimate differences in treatment effects.^23^ To the best of our knowledge, our study is the first meta-analysis in this area to use reconstructed individual participant data, which is considered to be the criterion standard for reporting survival data because it accounts for time-to-event data and censoring of events.^65,66^

### Limitations

We acknowledge the following limitations. All included studies were observational in nature, with consequent risk of selection bias, but we attempted to mitigate this by only including studies with propensity score matching, which reduces but does not completely remove selection bias.^29^ In addition, possible residual confounders such as degree of liver fibrosis, family history, viral genotype, adherence to antiviral therapy, and adherence to HCC surveillance^67^ were not assessed in the included studies. Given the challenges with conducting a randomized clinical trial in this topic, which would require an extremely large sample size of patients and long duration of follow-up owing to the low event rate, our study represents the most practical approach to minimize bias and to obtain robust estimates.

## Conclusions

In this meta-analysis, there was no clinically meaningful difference in the risk of HCC between tenofovir and entecavir. The RMST analysis, which accounts for varying estimated treatment effects across time for both the overall analysis and administrative database studies, determined that the difference in mean time to HCC in patients who received tenofovir vs patients who received entecavir was less than 3 weeks. There was no significant difference at the 3- and 5-year follow-ups between tenofovir and entecavir among clinical cohort studies, which may have had more detailed clinical data to adjust for confounders. The choice between tenofovir or entecavir should be decided based on patient-related factors, such as cost, convenience, availability, and tolerability.

## Notes

Supplement. **eMethods.** Search Strategy for Embase and Medline Databases **eTable 1.** Summary of Included Articles **eFigure 1.** Reconstructed Survival Curves **eFigure 2.** Assessment of Publication Bias **eTable 2.** Restricted Mean Survival Time (RMST) Analysis of HCC Incidence in Patients Treated With Tenofovir vs Entecavir **eFigure 3.** Cumulative Incidence of HCC in Patients Receiving Tenofovir vs Entecavir in Patients With Cirrhosis Only **eFigure 4.** Cumulative Incidence of HCC in Patients Receiving Tenofovir vs Entecavir in (A) Single Centre Studies and in (B) Multicentre Studies **eFigure 5.** Cumulative Incidence of HCC in Patients Receiving Tenofovir vs Entecavir in Studies Involving Treatment-Naïve Patients Only
eReferences.

## References

[1] GBD 2019 Diseases and Injuries Collaborators. Global burden of 369 diseases and injuries in 204 countries and territories, 1990-2019: a systematic analysis for the Global Burden of Disease Study 2019. Lancet. 2020;396(10258):1204-1222. doi:10.1016/S0140-6736(20)30925-9 33069326 PMC7567026

[2] World Health Organization Global Hepatitis Report. 2017. Accessed May 20, 2020. https://apps.who.int/iris/bitstream/handle/10665/255016/9789241565455-eng.pdf;jsessionid=6DB65DA61DB685B218A314037DBE0C09?sequence=1

[3] Udompap P, Kim WR. Development of hepatocellular carcinoma in patients with suppressed viral replication: changes in risk over time. Clin Liver Dis (Hoboken). 2020;15(2):85-90. doi:10.1002/cld.904 32226623 PMC7098665

[4] Huang DQ, Lim SG. Hepatitis B: who to treat? a critical review of international guidelines. Liver Int. 2020;40(suppl 1):5-14. doi:10.1111/liv.14365 32077616

[5] Chen CJ, Yang HI, Su J, ; REVEAL-HBV Study Group. Risk of hepatocellular carcinoma across a biological gradient of serum hepatitis B virus DNA level. JAMA. 2006;295(1):65-73. doi:10.1001/jama.295.1.65 16391218

[6] Papatheodoridis GV, Chan HLY, Hansen BE, Janssen HL, Lampertico P. Risk of hepatocellular carcinoma in chronic hepatitis B: assessment and modification with current antiviral therapy. J Hepatol. 2015;62(4):956-967. doi:10.1016/j.jhep.2015.01.002 25595883

[7] Varbobitis I, Papatheodoridis GV. The assessment of hepatocellular carcinoma risk in patients with chronic hepatitis B under antiviral therapy. Clin Mol Hepatol. 2016;22(3):319-326. doi:10.3350/cmh.2016.0045 27729632 PMC5066383

[8] Seto WK, Lau EH, Wu JT, . Effects of nucleoside analogue prescription for hepatitis B on the incidence of liver cancer in Hong Kong: a territory-wide ecological study. Aliment Pharmacol Ther. 2017;45(4):501-509. doi:10.1111/apt.13895 27976416

[9] Terrault NA, Lok ASF, McMahon BJ, . Update on prevention, diagnosis, and treatment of chronic hepatitis B: AASLD 2018 hepatitis B guidance. Hepatology. 2018;67(4):1560-1599. doi:10.1002/hep.29800 29405329 PMC5975958

[10] Sarin SK, Kumar M, Lau GK, . Asian-Pacific clinical practice guidelines on the management of hepatitis B: a 2015 update. Hepatol Int. 2016;10(1):1-98. doi:10.1007/s12072-015-9675-4 26563120 PMC4722087

[11] European Association for the Study of the Liver; European Association for the Study of the Liver. EASL 2017 Clinical Practice Guidelines on the management of hepatitis B virus infection. J Hepatol. 2017;67(2):370-398. doi:10.1016/j.jhep.2017.03.021 28427875

[12] Choi WM, Yip TCF, Lim YS, Wong GLH, Kim WR. Methodological challenges in meta-analysis to assess the risk of hepatocellular carcinoma between chronic hepatitis B treatments. J Hepatol. 2021;76(1):186-194. doi:10.1016/j.jhep.2021.09.017 34592365

[13] Zhang Z, Zhou Y, Yang J, Hu K, Huang Y. The effectiveness of TDF versus ETV on incidence of HCC in CHB patients: a meta analysis. BMC Cancer. 2019;19(1):511. doi:10.1186/s12885-019-5735-9 31142283 PMC6542001

[14] Choi WM, Choi J, Lim YS. Effects of Tenofovir vs Entecavir on Risk of Hepatocellular Carcinoma in Patients With Chronic HBV Infection: A Systematic Review and Meta-analysis. Clin Gastroenterol Hepatol. 2021;19(2):246-258.e9. doi:10.1016/j.cgh.2020.05.008 32407970

[15] Cheung KS, Mak LY, Liu SH, . Entecavir vs tenofovir in hepatocellular carcinoma prevention in chronic hepatitis B infection: a systematic review and meta-analysis. Clin Transl Gastroenterol. 2020;11(10):e00236. doi:10.14309/ctg.0000000000000236 33031195 PMC7544163

[16] Dave S, Park S, Murad MH, . Comparative effectiveness of entecavir versus tenofovir for preventing hepatocellular carcinoma in patients with chronic hepatitis B: a systematic review and meta-analysis. Hepatology. 2021;73(1):68-78. doi:10.1002/hep.31267 32277491 PMC8022893

[17] Li M, Lv T, Wu S, . Tenofovir versus entecavir in lowering the risk of hepatocellular carcinoma development in patients with chronic hepatitis B: a critical systematic review and meta-analysis. Hepatol Int. 2020;14(1):105-114. doi:10.1007/s12072-019-10005-0 31898210

[18] Gu L, Yao Q, Shen Z, . Comparison of tenofovir versus entecavir on reducing incidence of hepatocellular carcinoma in chronic hepatitis B patients: a systematic review and meta-analysis. J Gastroenterol Hepatol. 2020;35(9):1467-1476. doi:10.1111/jgh.15036 32180249

[19] Liu H, Shi Y, Hayden JC, Ryan PM, Rahmani J, Yu G. Tenofovir treatment has lower risk of hepatocellular carcinoma than entecavir treatment in patients with chronic hepatitis B: a systematic review and meta-analysis. Liver Cancer. 2020;9(4):468-476. doi:10.1159/000507253 32999872 PMC7506291

[20] Wang X, Liu X, Dang Z, . Nucleos(t)ide analogues for reducing hepatocellular carcinoma in chronic hepatitis B patients: a systematic review and meta-analysis. Gut Liver. 2020;14(2):232-247. doi:10.5009/gnl18546 31158948 PMC7096226

[21] Tseng CH, Hsu YC, Chen TH, . Hepatocellular carcinoma incidence with tenofovir versus entecavir in chronic hepatitis B: a systematic review and meta-analysis. Lancet Gastroenterol Hepatol. 2020;5(12):1039-1052. doi:10.1016/S2468-1253(20)30249-1 33007228

[22] Stewart LA, Tierney JF. To IPD or not to IPD? advantages and disadvantages of systematic reviews using individual patient data. Eval Health Prof. 2002;25(1):76-97. doi:10.1177/0163278702025001006 11868447

[23] Rulli E, Ghilotti F, Biagioli E, . Assessment of proportional hazard assumption in aggregate data: a systematic review on statistical methodology in clinical trials using time-to-event endpoint. Br J Cancer. 2018;119(12):1456-1463. doi:10.1038/s41416-018-0302-8 30420618 PMC6288087

[24] Page MJ, McKenzie JE, Bossuyt PM, . The PRISMA 2020 statement: an updated guideline for reporting systematic reviews. BMJ. 2021;372(71):n71. doi:10.1136/bmj.n71 33782057 PMC8005924

[25] Stewart LA, Clarke M, Rovers M, ; PRISMA-IPD Development Group. Preferred Reporting Items for Systematic Review and Meta-Analyses of individual participant data: the PRISMA-IPD Statement. JAMA. 2015;313(16):1657-1665. doi:10.1001/jama.2015.3656 25919529

[26] Syn NL, Kabir T, Koh YX, . Survival advantage of laparoscopic versus open resection for colorectal liver metastases: a meta-analysis of individual patient data from randomized trials and propensity-score matched studies. Ann Surg. 2020;272(2):253-265. doi:10.1097/SLA.0000000000003672 32675538

[27] Syn NL, Cummings DE, Wang LZ, . Association of metabolic-bariatric surgery with long-term survival in adults with and without diabetes: a one-stage meta-analysis of matched cohort and prospective controlled studies with 174 772 participants. Lancet. 2021;397(10287):1830-1841. doi:10.1016/S0140-6736(21)00591-2 33965067

[28] Ioannidis JP, Haidich AB, Pappa M, . Comparison of evidence of treatment effects in randomized and nonrandomized studies. JAMA. 2001;286(7):821-830. doi:10.1001/jama.286.7.821 11497536

[29] Yao XI, Wang X, Speicher PJ, . Reporting and guidelines in propensity score analysis: a systematic review of cancer and cancer surgical studies. J Natl Cancer Inst. 2017;109(8):djw323. doi:10.1093/jnci/djw323 28376195 PMC6059208

[30] Grose E, Wilson S, Barkun J, . Use of propensity score methodology in contemporary high-impact surgical literature. J Am Coll Surg. 2020;230(1):101-112.e2. doi:10.1016/j.jamcollsurg.2019.10.003 31672675

[31] Guyot P, Ades AE, Ouwens MJNM, Welton NJ. Enhanced secondary analysis of survival data: reconstructing the data from published Kaplan-Meier survival curves. BMC Med Res Methodol. 2012;12(1):9. doi:10.1186/1471-2288-12-9 22297116 PMC3313891

[32] Ahmad N, Ahuja SD, Akkerman OW, ; Collaborative Group for the Meta-Analysis of Individual Patient Data in MDR-TB treatment–2017. Treatment correlates of successful outcomes in pulmonary multidrug-resistant tuberculosis: an individual patient data meta-analysis. Lancet. 2018;392(10150):821-834. doi:10.1016/S0140-6736(18)31644-1 30215381 PMC6463280

[33] Gaudry S, Hajage D, Benichou N, . Delayed versus early initiation of renal replacement therapy for severe acute kidney injury: a systematic review and individual patient data meta-analysis of randomised clinical trials. Lancet. 2020;395(10235):1506-1515. doi:10.1016/S0140-6736(20)30531-6 32334654

[34] de Jong VMT, Moons KGM, Riley RD, . Individual participant data meta-analysis of intervention studies with time-to-event outcomes: a review of the methodology and an applied example. Res Synth Methods. 2020;11(2):148-168. doi:10.1002/jrsm.1384 31759339 PMC7079159

[35] Debray TP, Moons KG, Ahmed I, Koffijberg H, Riley RD. A framework for developing, implementing, and evaluating clinical prediction models in an individual participant data meta-analysis. Stat Med. 2013;32(18):3158-3180. doi:10.1002/sim.5732 23307585

[36] Smith CT, Williamson PR, Marson AG. Investigating heterogeneity in an individual patient data meta-analysis of time to event outcomes. Stat Med. 2005;24(9):1307-1319. doi:10.1002/sim.2050 15685717

[37] Royston P, Parmar MKB. An approach to trial design and analysis in the era of non-proportional hazards of the treatment effect. Trials. 2014;15(1):314. doi:10.1186/1745-6215-15-314 25098243 PMC4133607

[38] Dehbi HM, Royston P, Hackshaw A. Life expectancy difference and life expectancy ratio: two measures of treatment effects in randomised trials with non-proportional hazards. BMJ. 2017;357:j2250. doi:10.1136/bmj.j2250 28546261 PMC5444092

[39] Royston P, Parmar MK. The use of restricted mean survival time to estimate the treatment effect in randomized clinical trials when the proportional hazards assumption is in doubt. Stat Med. 2011;30(19):2409-2421. doi:10.1002/sim.4274 21611958

[40] DerSimonian R, Laird N. Meta-analysis in clinical trials. Control Clin Trials. 1986;7(3):177-188. doi:10.1016/0197-2456(86)90046-2 3802833

[41] Clopper CJ, Pearson ES. The use of confidence or fiducial limits illustrated in the case of the binomial. Biometrika. 1934;26(4):404-413. doi:10.1093/biomet/26.4.404

[42] Schwarzer G, Chemaitelly H, Abu-Raddad LJ, Rücker G. Seriously misleading results using inverse of Freeman-Tukey double arcsine transformation in meta-analysis of single proportions. Res Synth Methods. 2019;10(3):476-483. doi:10.1002/jrsm.1348 30945438 PMC6767151

[43] Wells GA, Shea B, O'Connell D, et al. The Newcastle-Ottawa Scale (NOS) for assessing the quality of nonrandomised studies in meta-analyses. The Ottawa Hospital Research Institute. 2014. Accessed November 12, 2021. http://www.ohri.ca/programs/clinical_epidemiology/oxford.asp

[44] Egger M, Davey Smith G, Schneider M, Minder C. Bias in meta-analysis detected by a simple, graphical test. BMJ. 1997;315(7109):629-634. doi:10.1136/bmj.315.7109.629 9310563 PMC2127453

[45] Choi J, Kim HJ, Lee J, Cho S, Ko MJ, Lim YS. Risk of hepatocellular carcinoma in patients treated with entecavir vs tenofovir for chronic hepatitis B: a Korean nationwide cohort study. JAMA Oncol. 2019;5(1):30-36. Retracted and replaced in: *JAMA Oncol*. 2019;5(6):913-914. doi:10.1001/jamaoncol.2018.4070 30267080 PMC6439769

[46] Kim SU, Seo YS, Lee HA, . A multicenter study of entecavir vs tenofovir on prognosis of treatment-naïve chronic hepatitis B in South Korea. J Hepatol. 2019;71(3):456-464. doi:10.1016/j.jhep.2019.03.028 30959156

[47] Lee SW, Kwon JH, Lee HL, . Comparison of tenofovir and entecavir on the risk of hepatocellular carcinoma and mortality in treatment-naïve patients with chronic hepatitis B in Korea: a large-scale, propensity score analysis. Gut. 2020;69(7):1301-1308. doi:10.1136/gutjnl-2019-318947 31672838 PMC7306978

[48] Ha I, Chung JW, Jang ES, Jeong SH, Kim JW. Comparison of the on-treatment risks for hepatocellular carcinoma between entecavir and tenofovir: a propensity score matching analysis. J Gastroenterol Hepatol. 2020;35(10):1774-1781. doi:10.1111/jgh.15031 32154938

[49] Kim BG, Park NH, Lee SB, . Mortality, liver transplantation and hepatic complications in patients with treatment-naïve chronic hepatitis B treated with entecavir vs tenofovir. J Viral Hepat. 2018;25(12):1565-1575. doi:10.1111/jvh.12971 29998592

[50] Oh H, Yoon EL, Jun DW, ; Long-Term Safety of Entecavir and Tenofovir in Patients With Treatment-Naive Chronic Hepatitis B Virus (CHB) Infection (SAINT) Study. No difference in incidence of hepatocellular carcinoma in patients with chronic hepatitis B virus infection treated with entecavir vs tenofovir. Clin Gastroenterol Hepatol. 2020;18(12):2793-2802.e6. doi:10.1016/j.cgh.2020.02.046 32135246

[51] Shin JW, Jeong J, Jung SW, . Comparable incidence of hepatocellular carcinoma in chronic hepatitis B patients treated with entecavir or tenofovir. Dig Dis Sci. 2021;66(5):1739-1750. doi:10.1007/s10620-020-06375-3 32524416

[52] Ha Y, Chon YE, Kim MN, Lee JH, Hwang SG. Hepatocellular carcinoma and death and transplantation in chronic hepatitis B treated with entecavir or tenofovir disoproxil fumarate. Sci Rep. 2020;10(1):13537. doi:10.1038/s41598-020-70433-z 32782369 PMC7419516

[53] Chang TS, Yang YH, Chen WM, . Long-term risk of primary liver cancers in entecavir versus tenofovir treatment for chronic hepatitis B. Sci Rep. 2021;11(1):1365. doi:10.1038/s41598-020-80523-7 33446835 PMC7809351

[54] Hu TH, Yueh-Hsia Chiu S, Tseng PL, . Five-year comparative risk of hepatocellular carcinoma development under entecavir or tenofovir treatment-naïve patients with chronic hepatitis B–related compensated cirrhosis in Taiwan. Aliment Pharmacol Ther. 2020;52(11-12):1695-1706. 33111400 10.1111/apt.16116

[55] Chen CH, Chen CY, Wang JH, . Comparison of incidence of hepatocellular carcinoma between chronic hepatitis B patients with cirrhosis treated with entecavir or tenofovir in Taiwan: a retrospective study. Am J Cancer Res. 2020;10(11):3882-3895. 33294274 PMC7716174

[56] Yip TCF, Wong VWS, Chan HLY, Tse YK, Lui GCY, Wong GLH. Tenofovir is associated with lower risk of hepatocellular carcinoma than entecavir in patients with chronic HBV infection in China. Gastroenterology. 2020;158(1):215-225. doi:10.1053/j.gastro.2019.09.025 31574268

[57] Su F, Berry K, Ioannou GN. No difference in hepatocellular carcinoma risk between chronic hepatitis B patients treated with entecavir versus tenofovir. Gut. 2021;70(2):370-378. 32229544 10.1136/gutjnl-2019-319867

[58] Hsu YC, Wong GLH, Chen CH, . Tenofovir versus entecavir for hepatocellular carcinoma prevention in an international consortium of chronic hepatitis B. Am J Gastroenterol. 2020;115(2):271-280. doi:10.14309/ajg.0000000000000428 31634265

[59] Lee HW, Cho YY, Lee H, . Impact of tenofovir alafenamide vs. entecavir on hepatocellular carcinoma risk in patients with chronic hepatitis B. Hepatol Int. 2021;15(5):1083-1092. doi:10.1007/s12072-021-10234-2 34402025

[60] Woo G, Tomlinson G, Nishikawa Y, . Tenofovir and entecavir are the most effective antiviral agents for chronic hepatitis B: a systematic review and Bayesian meta-analyses. Gastroenterology. 2010;139(4):1218-1229. doi:10.1053/j.gastro.2010.06.042 20600036

[61] Gao L, Trinh HN, Li J, Nguyen MH. Tenofovir is superior to entecavir for achieving complete viral suppression in HBeAg-positive chronic hepatitis B patients with high HBV DNA. Aliment Pharmacol Ther. 2014;39(6):629-637. doi:10.1111/apt.12629 24467455 PMC3999385

[62] Ma X, Liu S, Wang M, . Tenofovir alafenamide fumarate, tenofovir disoproxil fumarate and entecavir: which is the most effective drug for chronic hepatitis B? a systematic review and meta-analysis. J Clin Transl Hepatol. 2021;9(3):335-344. doi:10.14218/JCTH.2020.00164 34221919 PMC8237148

[63] Tenney DJ, Rose RE, Baldick CJ, . Long-term monitoring shows hepatitis B virus resistance to entecavir in nucleoside-naïve patients is rare through 5 years of therapy. Hepatology. 2009;49(5):1503-1514. doi:10.1002/hep.22841 19280622

[64] Marcellin P, Gane E, Flisiak R, . Long term treatment with tenofovir disoproxil fumarate for chronic hepatitis B infection is safe and well tolerated and associated with durable virologic response with no detectable resistance: 8 year results from two phase 3 trials. Paper presented at: 65th Annual Meeting of the American Association for the Study of Liver Disease; November 7-11, 2014; Boston, Massachusetts.

[65] Riley RD, Lambert PC, Abo-Zaid G. Meta-analysis of individual participant data: rationale, conduct, and reporting. BMJ. 2010;340:c221. doi:10.1136/bmj.c221 20139215

[66] Tan DJH, Lim WH, Yong JN, . UNOS down-staging criteria for liver transplantation of hepatocellular carcinoma: systematic review and meta-analysis of 25 studies. Clin Gastroenterol Hepatol. Published online February 15, 2022. doi:10.1016/j.cgh.2022.02.018 35181565

[67] Tan DJH, Ng CH, Lin SY, . Clinical characteristics, surveillance, treatment allocation, and outcomes of non-alcoholic fatty liver disease-related hepatocellular carcinoma: a systematic review and meta-analysis. Lancet Oncol. 2022;23(4):521-530. doi:10.1016/S1470-2045(22)00078-X 35255263 PMC9718369

