# Supplementary material for: Risk of Hepatocellular Carcinoma With Tenofovir vs Entecavir Treatment for Chronic Hepatitis B Virus: A Reconstructed Individual Patient Data Meta-analysis
Source: JAMA Netw Open. 2022 Jun 29;5(6):e2219407. doi: 10.1001/jamanetworkopen.2022.19407 (PMC9244612; doi:10.1001/jamanetworkopen.2022.19407)
Supplement: Supplement. — eMethods. Search Strategy for Embase and Medline Databases eTable 1. Summary of Included Articles eFigure 1. Reconstructed Survival Curves eFigure 2. Assessment of Publication Bias eTable 2. Restricted Mean Survival Time (RMST) Analysis of HCC Incidence in Patients Treated With Tenofovir vs Entecavir eFigure 3. Cumulative Incidence of HCC in Patients Receiving Tenofovir vs Entecavir in Patients With Cirrhosis Only eFigure 4. Cumulative Incidence of HCC in Patients Receiving Tenofovir vs Entecavir in (A) Single Centre Studies and in (B) Multicentre Studies eFigure 5. Cumulative Incidence of HCC in Patients Receiving Tenofovir vs Entecavir in Studies Involving Treatment-Naïve Patients Only eReferences. [file jamanetwopen-e2219407-s001.pdf]

Supplementary Online Content

Tan DJH, Ng CH, Tay PWL, et al. Risk of hepatocellular carcinoma with tenofovir vs entecavir treatment for chronic hepatitis B virus: a reconstructed individual patient data meta-analysis. *JAMA Netw Open*. 2022;5(6):e2219407. doi:10.1001/jamanetworkopen.2022.19407

eMethods. Search Strategy for Embase and Medline Databases

eTable 1. Summary of Included Articles

eFigure 1. Reconstructed Survival Curves

eFigure 2. Assessment of Publication Bias

eTable 2. Restricted Mean Survival Time (RMST) Analysis of HCC Incidence in Patients Treated With Tenofovir vs Entecavir

eFigure 3. Cumulative Incidence of HCC in Patients Receiving Tenofovir vs Entecavir in Patients With Cirrhosis Only

eFigure 4. Cumulative Incidence of HCC in Patients Receiving Tenofovir vs Entecavir in (A) Single Centre Studies and in (B) Multicentre Studies

eFigure 5. Cumulative Incidence of HCC in Patients Receiving Tenofovir vs Entecavir in Studies Involving Treatment-Naïve Patients Only

References.

This supplementary material has been provided by the authors to give readers additional information about their work.

©2022 Tan DJH et al. *JAMA Network Open*.

eMethods. Search Strategy for Embase and Medline Databases

Embase

1: ('liver neoplasm*' or 'liver cancer*' or 'hepatic neoplasm*' or 'hepatic cancer*' or 'hepatocellular carcinoma' or 'hepatocellular cancer*' or hcc):ti,ab or 'liver neoplasms'/exp

2: 'Hepatitis B'/exp or ('Hepatitis B' or HBV):ti,ab

3: (entecavir or tenofovir or (nucleoside or nucleotide)):ti,ab

4: 1 and 2 and 3

Medline

1: ('liver neoplasm*' or 'liver cancer*' or 'hepatic neoplasm*' or 'hepatic cancer*' or 'hepatocellular carcinoma' or 'hepatocellular cancer*' or hcc).ti,ab. or exp liver neoplasms/

2: exp Hepatitis B/ or ('Hepatitis B' or HBV).ti,ab.

3: (entecavir or tenofovir or (nucleoside or nucleotide)).ti,ab.

4: 1 and 2 and 3

©2022 Tan DJH et al. *JAMA Network Open*.

eTable 1. Summary of Included Articles

Name, year	Country or Region	Study Scale	Drug	Number of Participants	Mean or Median age, years	Male Participants (%)	Female Participants (%)	Previous nucleos(t)ide analogue treatment (%)	Cirrhosis (%)	Follow up, months (median (IQR) or mean (SD))	HCC occurrence	Factors included in PS model	Results reported in study (Entecavir as reference group)	NOS score
Hsu et al,[1] 2020	USA and Asia	Multicentre; international	Entecavir vs tenofovir disoproxil fumarate	Before matching: (4837 vs 700); After matching: (520 vs 520)	Before matching: 50.8 (0.2) vs 45.7 (0.5); After matching: 44.1 (0,5) vs 44.9 (0.6)	Before matching: 3328 (68.8%) vs 456 (65·1%); After matching: 354 (68·1%) vs 338 (65·0%)	Before matching: 1509 (31.2%) vs 244 (34.9%); After matching: 166 (31.9%) vs 182 (35.0%)	Naive	Before matching: 1344 (27·8%) vs 131 (18·7%); After matching: 107 (20·6%) vs 105 (20·2%)	Before matching: Median 60 (39.6-60) vs 38·7 (23.8-56.2); After matching: 60 (36.5-60) vs 38·9 (23.9-57.7)	Before matching: 285 vs 13; After matching:19 vs 11	Age, Gender, Asian countries, HbeAg, ALT, HBV DNA, Platelet Count, Cirrhosis, DM, Hepatic Decompensation	crude HR: 0.77 (0.37 - 1.60); p=0.48<br><br>adjusted HR: 0.81 (0.42 - 1.56); p=0.52	9
Choi et al,[2] 2018	South Korea	Single centre (hospital cohort);<br><br>Multicentre (national claims database cohort) from a single country	Entecavir vs tenofovir disoproxil fumarate	Single centre before matching: (1560 vs 1141); After matching: (869 vs 869);<br><br>Multicentre before matching: (11 464 vs 12692); After matching: (10923 vs 10923)	Single centre before matching: 49·2 (10·5) vs 48·1 (10·5); After matching: 48·8 (10·4) vs 48·8 (10·4);<br><br>Multicentre before matching: 49·3 (9·8) vs 48·6 (9·8); After matching: 49·1 (9·8) vs 49 (9·8)	Single centre before matching: 965 (61·9%) vs 962 (60·6%); After matching: 519 (59·7%) vs 540 (62·1%);<br><br>Multicentre before matching: 7171 (62·6%) vs 7949 (62·6%); After matching: 6802 (62·3%) vs 6834 (62·6%)	Single centre before matching: 595 (38·1%) vs 179 (39·4%); After matching: 350 (40·3%) vs 329 (37·9%);<br><br>Multicentre before matching: 4293(37·4%) vs 4743 (37·4%); After matching: 4121 (37·7%) vs 4089 (37·4%)	Naive	Single centre before matching: 935 (59·3%) vs 653 (57·2%); After matching: 511 (58·8%) vs 505 (58·1%);<br><br>Multicentre before matching: 2991 (26·1%) vs 3488 (27·5%); After matching: 2891 (26·5%) vs 2919 (26·7%)	Single centre before matching: Median 48 (36-48) vs 32(23-43); After matching: 48 (35-48) vs 32 (22-44);<br><br>Multicentre before matching: 51 (40.4-55.4) vs 36 (29.3-42.1); After matching: 51 (40.6-55.4) vs 36 (29.2-42.1)	Single centre before matching: 115 vs 39; After matching: 61 vs 31;<br><br>Multicentre before matching: 590 vs 394; After matching: 567 vs 350	Age, Gender HBeAg, HBV DNA, ALT, Albumin, Bilirubin, INR, Platelet Count, Creatinine, DM, HTN, Cirrhosis, Ascites, Child-Pugh score; Chinese University HCC score, Cirrhosis–HCC score	adjusted HR: 0.68 (0.46 - 0.99); p=0.04	9
Kim et al,[3] 2019	South Korea	Multicentre; single country	Entecavir vs tenofovir	Before matching: (1484 vs	Before matching: 48·2	Before matching: 889 (59·9%)	Before matching: 595 (40·1%) vs	Naive	Before matching:	Before matching:	Before matching: 138 vs 102	Age, Gender, DM, HTN, Cirrhosis, HBeAg	adjusted HR: 1.02 (0.77 -	8

©2022 Tan DJH et al. *JAMA Network Open*.

			disoproxil fumarate	1413); After matching: (1278 vs 1278)	(11·5) vs 48·8 (12·0); After matching: 48·6 (11·4) vs 48·2 (12·0)	vs 913 (64·6%); After matching: 793 (62·1%) vs 794 (62·1%)	500 (35·4%); After matching: 485 (37·9%) vs 484 (37·9%)		499 (33·6%) vs 411 (29·1%); After matching: 394 (30·8%) vs 400 (31·3%)	Mean 58.1 vs 51.3		status, Bilirubin, Albumin, Platelet Count	1.35); p=0.88	
Lee et al,[4] 2020	South Korea	Single centre; single country	Entecavir vs tenofovir disoproxil fumarate	Before matching: (1583 vs 1439); After matching: (1370 vs 1370)	Before matching: 46·7 (11·8) vs 47·3 (11·6); After matching: 47·0 (11·7) vs 46·9 (11·3)	Before matching: 926 (58·5%) vs 841 (58·4%); After matching: 806 (58·8%) vs 798 (58·3%)	Before matching: 657 (41·5%) vs 598 (41·6%); After matching: 564 (41·2%) vs 572 (41·7%)	Naive	Before matching: 567 (35·8%) vs 483 (33·6%); After matching: 465 (33·9%) vs 464 (33·9%)	Before matching: Mean 51·5 vs 36·6; 51·5 vs 36·6	Before matching: 84 vs 50; After matching: 64 vs 47	Age, Gender, Liver Disease Severity, APRI, FIB-4 index, DM, HTN, BMI, Alcohol Intake, Oesophageal varix, AST, ALT, Bilirubin, Albumin, Creatinine, Gamma-glutamyl Transferase, PT, Platelet Count, Child-Pugh score, HBeAg status, HBV DNA and AFP	crude HR: 1.03 (0.70 - 1.51); p=0.88<br><br>adjusted HR: 1.08 (0.52 - 2.24); p=0.84	9
Ha et al,[5] 2020	South Korea	Single centre; single country	Entecavir vs tenofovir disoproxil fumarate	Before matching: (921 vs 419); After matching: (415 vs 415)	Before matching: 48 (15·0) vs 45 (16·0); After matching: 48 (16·0) vs 48 (14·0)	Before matching: 558 (61·0%) vs 266 (63·0%); After matching: 181 (61·0%) vs 179 (60·0%)	Before matching: 363 (39·0%) vs 153 (37·0%); After matching: 117 (39·0%) vs 119 (40·0%)	Naive	Before matching: 259 (28%) vs 39 (9·3%);After matching: 39 (9%) vs 39 (9%)	-	Before matching: 82 vs 24	Age, Gender, Alcohol Intake, DM, Cirrhosis, HBeAg, HBV DNA, HBsAg titer, AST, ALT, AFP, Albumin, Bilirubin, PT, Platelet Count, Treatment Initiation	adjusted HR: 1.84 (0.90 - 3.79); p=0.088	8
Kim et al,[6] 2018	South Korea	Single centre; single country	Entecavir vs tenofovir disoproxil fumarate	Before matching: (721 vs 604); After matching: (354 vs 354)	Before matching: 52 (11) vs 50 (11); After matching: 51 (11) vs 51 (11)	Before matching: 471 (65·3%) vs 363 (60·1%); After matching: 220 (62·1%) vs 222 (62·7%)	Before matching: 250 (34·7%) vs 241 (39·9%); After matching: 134 (37·9%) vs 132 (37·3%)	Naive	Before matching: 346 (48%) vs 267 (44·2%); After matching: 169 (47·7%) vs 156 (44·1%)	Before matching: Median 66 (36-88) vs 33(21-46);After matching: Mean 48·1 vs 32·9	Before matching: 40 vs 14; After matching: 24vs7	Age, Gender, HBeAg, Cirrhosis, HBV DNA, ALT, AST, Albumin, Bilirubin, Creatinine, INR, Platelet Count, DM, HTN	crude HR: 0.53 (0.22 - 1.25); p=0.15<br><br>adjusted HR: 0.56 (0.21 - 1.54); p=0.27	8

©2022 Tan DJH et al. *JAMA Network Open*.

Oh et al,[7] 2020	South Korea	Multicentre; single country	Entecavir vs tenofovir disoproxil fumarate	Before matching: (753 vs 807); After matching: (516 vs 516)	Before matching: 48·7 (11·4) vs 46·3 (11·2); After matching: 49·2 (12·6) vs 49 (9·4)	Before matching: 480 (63·7%) vs 503 (62·3%); After matching: 319 (61·8%) vs 325 (63·0%)	Before matching: 273 (36·3%) vs 304 (37·7%); After matching: 197 (38·2%) vs 191 (37·0%)	Naive	Before matching: 315 (41·8%) vs 310 (38·4%); After matching: 238 (46·1%) vs 224 (43·4%)	Before matching: Mean 56·4 (12) vs 54(13.2); After matching: 58·8(10.8) vs 56·4(12)	Before matching: 34 vs 45; After matching: 29 vs 37	Age, Gender, CKD, HTN, DM, Cirrhosis, Decompensation, HBeAg, HBV DNA, Child–Pugh Score, MELD, FIB-4, AFP, Platelet count, Albumin, Bilirubin, eGFR, INR	crude HR: 1.30 (0.80 - 2.02); p=0.30	8
Yip et al,[8] 2020	Hong Kong	Multicentre; single territory; electronic health record	Entecavir vs tenofovir disoproxil fumarate	Before matching: (28 041 vs 1309); After matching: (1200 vs 1200)	Before matching: 53·4 (13·0) vs 43·2 (13·1); After matching: 42·9 (12·7) vs 44·4 (13·1)	Before matching: 18 094 (64·5%) vs 591 (45·1%); After matching: 2267 (48·9%) vs 587 (48·9%)	Before matching: 9947 (35·5%) vs 718 (54·9%); After matching: 2369 (51·1%) vs 613 (51·1%)	Naive	Before matching: 3822 (13·6%) vs 38 (2·9%); After matching: 167 (3·6%) vs 37(3·1%)	After matching: 33·6(18-55.2) vs 34·8 (18-54)	Before matching: 1386 vs 8; After matching: 76 vs 8	Age, gender, Serum HBV DNA, ALT, Albumin, Total bilirubin, INR, Platelet count, Creatinine, RRT, Cirrhosis, Ascites, Hepatic Encephalopathy, DM, HTN, Treatment Initiation	adjusted HR: 0.39 (0.18 - 0.84); p=0.016	9
Shin et al,[9] 2021	South Korea	Single centre; single country	Entecavir vs tenofovir disoproxil fumarate	Before matching: (894 vs 900); After matching: (589vs 589)	Before matching: 52.0 (11.0) vs 51.0 (11.0); After matching: 50.0 (11.0) vs 50.0 (11.0)	Before matching: 597 (66.78) vs 571 (63.44); After matching: 365 (61.97) vs 358 (60.78)	Before matching: 297 (33.2) vs 329 (36.6); After matching: 224 (38.0) vs 231 (39.2)	Naive	Before matching: 440 (49.22) vs 375 (41.67); After matching: 276 (46.86) vs 282 (47.88)	Before matching: Median 82.8 (51.6-105.7) vs 45.6 (32.4-60)	Before matching:31 vs 74	Age, Gender, HBV DNA, HBeAg, ALT, Albumin, INR, Platelet Count, DM, Cirrhosis, Virologic Response	adjusted HR: 0.77 (0.46 - 1.29); p=0.32	
Chen et al,[10] 2020	Taiwan	Multicentre; single country	Entecavir vs tenofovir disoproxil fumarate	545 vs 545	55.4 (11.7) vs 54.5 (12.9)	721 (72.6) vs 428 (75.5)	272 (27.4) vs 139 (24.5)	11.9% vs 15.7%*	545 (100.0) vs 545 (100.0)	-	196 vs 48	Age, Gender, DM, HTN, Decompensation, AST, ALT, Bilirubin, INR, Albumin, Platelet Count, eGFR, FIB-4, APRI, HBV DNA, HBeAg, AFP	adjusted HR: 0.66 (0.46 - 0.95); p=0.023	9
Hu et al,[11] 2020	Taiwan	Single centre; single country	Entecavir vs tenofovir disoproxi	Before matching: (678 vs 216); After	Before matching: 59.4 (11.1) vs 56.1 (11.6);	Before matching: 491 (72.4) vs 162 (75.0); After	Before matching: 187 (27.6) vs 54 (25.0); After matching: 165	Naive	Before matching: 678 (100.0) vs 216 (100.0);	-	Before matching: 111 vs 19	Age, AFP, Albumin, HBV DNA, eGFR, family history, Upper	adjusted HR: 0.66 (0.38 - 1.14); p=0.14	8

©2022 Tan DJH et al. *JAMA Network Open*.

			l fumarate	matching: (607 vs 157)	After matching: 58.8 (10.8) vs 58.6 (11.0)	matching: 442 (72.8) vs 115 (73.2)	(27.2) vs 42 (26.8)		After matching: 607 (100.0) vs 157 (100.0)			Gastrointestinal Varices		
Ha et al,[12] 2020	South Korea	Single centre; single country	Entecavir vs tenofovir disoproxi l fumarate	180 vs 224	45.5 (10.8) vs 44.5 (11.4)	106 (58.9) vs 120 (53.6)	74 (41.1) vs 104 (46.4)	Naive	67 (37.20 vs 78 (34.8)	Median 64.0 (30.5-84.3) vs 49.1 (37.7-62.2)	18 vs 6	Age, Gender, DM, HTN, Cirrhosis, HBeAg, AST, ALT, Bilirubin, Albumin, Platelet Count	crude HR: 0.27 (0.08 - 0.98); p=0.046	9
Chang et al,[13] 2021	Taiwan	Multicentre; single country	Entecavir vs tenofovir disoproxi l fumarate	Before matching: (5348 vs 1900); After matching (3304 vs 1652)	Before matching: 54.0 (11.92) vs 51.0 (12.19); After matching: 52.0 (11.0) vs 52.0 (12.0)	Before matching: 3544 (66.0) vs 1302 (69.0); After matching: 2210 (67.0) vs 1106 (67.0)	Before matching: 1804 (34.0) vs 598 (31.0); After matching:1094 (33.0) vs 546 (33.0)	Before matching: 9.0% vs 23.0%; After matching: 8.0% vs 23.0%*	Before matching: 1590 (30.0) vs 590 (31.0); After matching: 930 (28.0) vs 509 (31.0)	Before matching: Mean 39.6 (24.6) vs 40.08 (22.1); After matching: 40.08(24.4) vs 41.04(22.2)	Before matching: 375 vs 100	Age, Race, Gender, BMI, DM, CCI, alcoholism, substance abuse, HIV, HCV, Cirrhosis, HBV, HBeAg, Platelet Count, AST/ALT, Albumin, Bilirubin, INR, Creatinine	crude HR: 0.82 (0.64 - 1.04); p=0.10<br><br>adjusted HR: 0.83 (0.65 - 1.06); p=0.13	9
Su et al,[14] 2021	USA	Multicentre; single country	Entecavir vs tenofovir disoproxi l fumarate	2193 vs 1094	56.5 (12.2) vs 55.4 (12.4)	2116 (96.5) vs 1039 (95.0)	77 (3.5) vs 60 (5.0)	36.6% vs 31.9%*	453 (20.7) vs 227 (20.8)	Mean 67.2 vs 64.8	167 vs 85		crude HR: 1.10 (0.83 - 1.43)<br><br>adjusted HR: 1.00 (0.76 - 1.32)	8

Abbreviations: IQR = interquartile range; SD = standard deviation; HBV = hepatitis B virus; INR = international normalised ratio; HTN = hypertension, DM = diabetes mellitus; RRT = renal replacement therapy; HbeAg = Hepatitis B e antigen; ALT = alanine aminotransferase; AST = aspartate aminotransferase; AFP = alpha fetoprotein; APRI = AST to platelet ratio index; BMI = body mass index; FIB-4 = fibrosis-4 index; PT = prothrombin time; MELD = model for end-stage liver disease; eGFR = estimated glomerular filtration rate; CCI = Charlson Comorbidity Index; HIV = human immunodeficiency virus; NOS = Newcastle-Ottawa Score; PS = propensity score; HR = hazard ratio

*Refers to the proportion of patients that had received previous nucleos(t)ide analogue treatment before initiation of tenofovir or entecavir

©2022 Tan DJH et al. *JAMA Network Open*.

eFigure 1. Reconstructed Survival Curves

Hsu et al 2020

Original

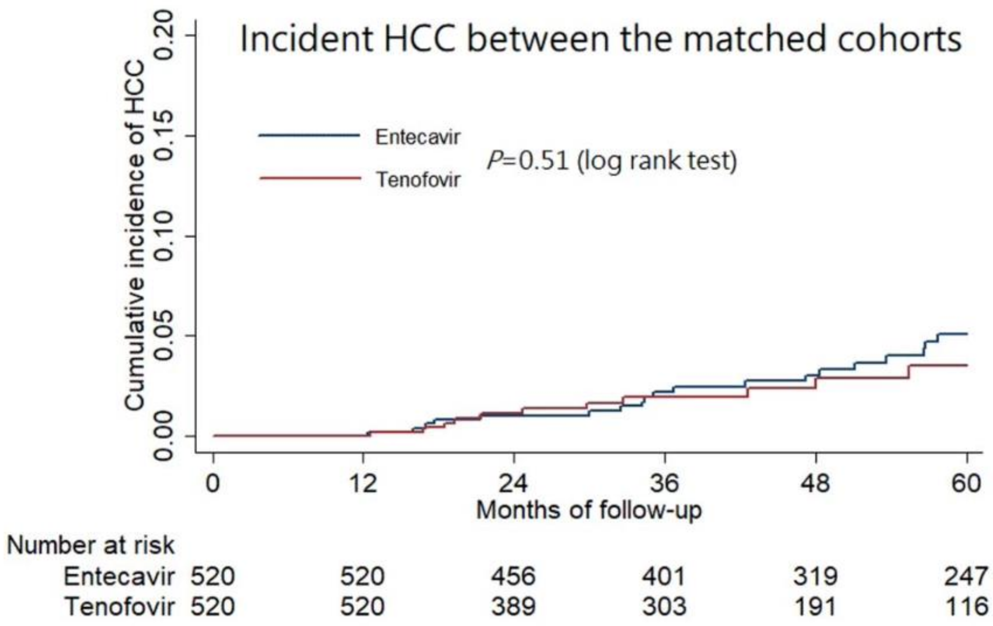

Reconstructed

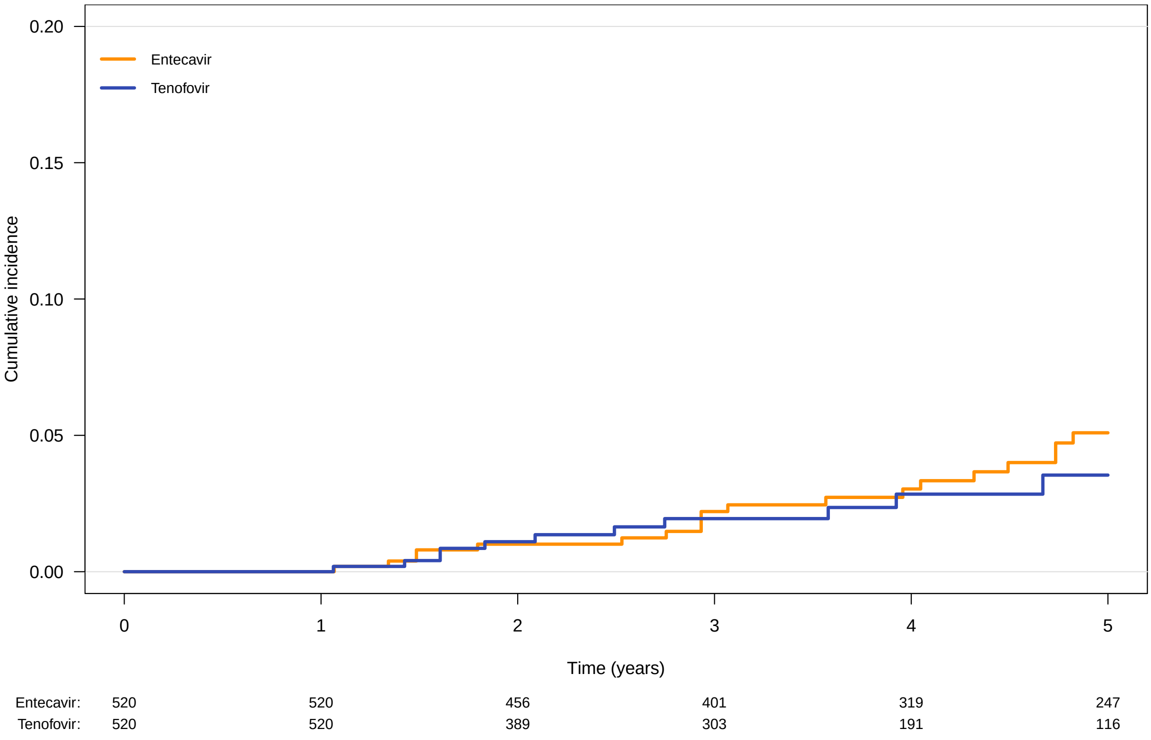

©2022 Tan DJH et al. *JAMA Network Open*.

Choi et al 2018

Original

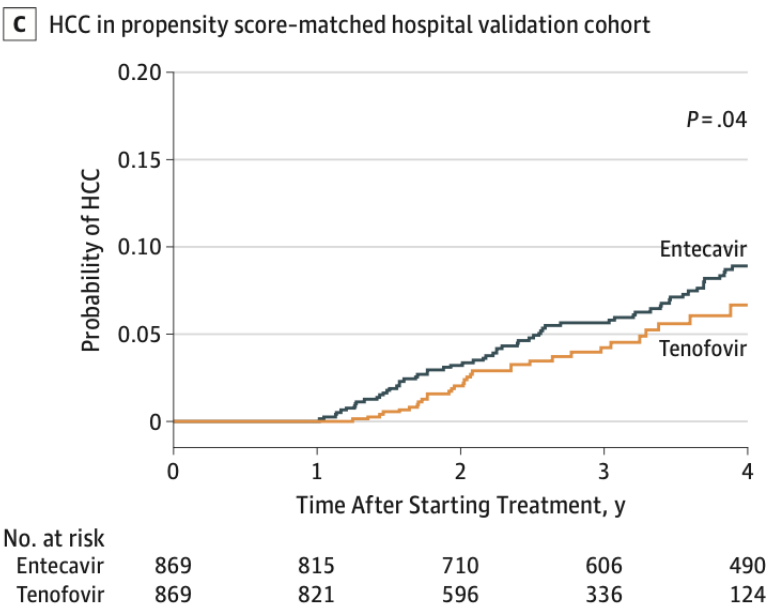

Reconstructed

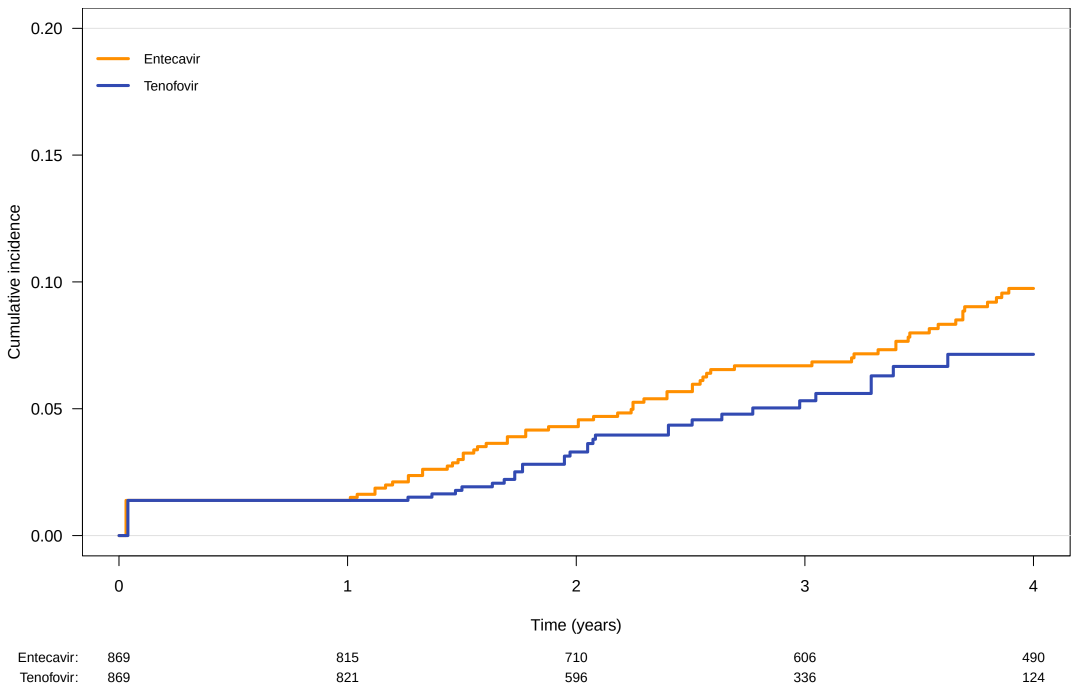

©2022 Tan DJH et al. *JAMA Network Open*.

Kim et al 2019

Original

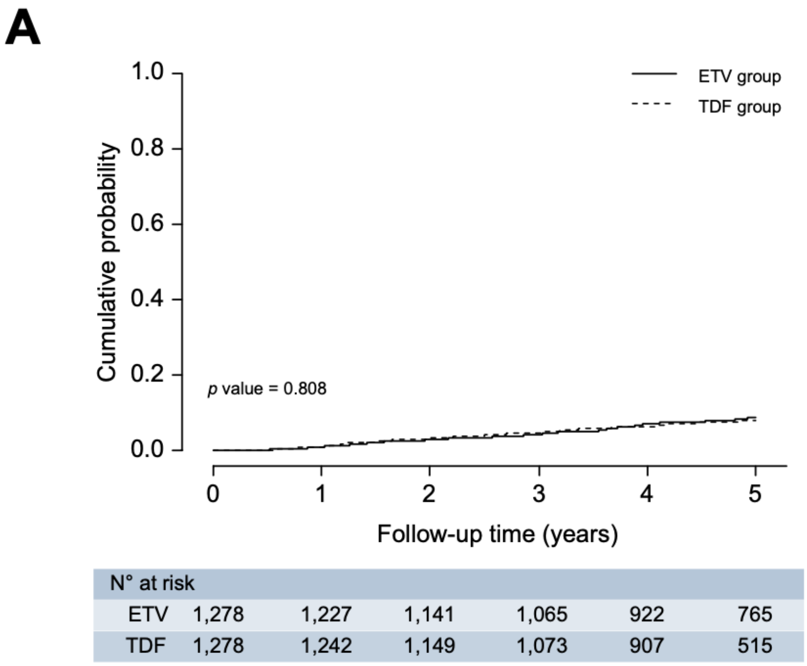

Reconstructed

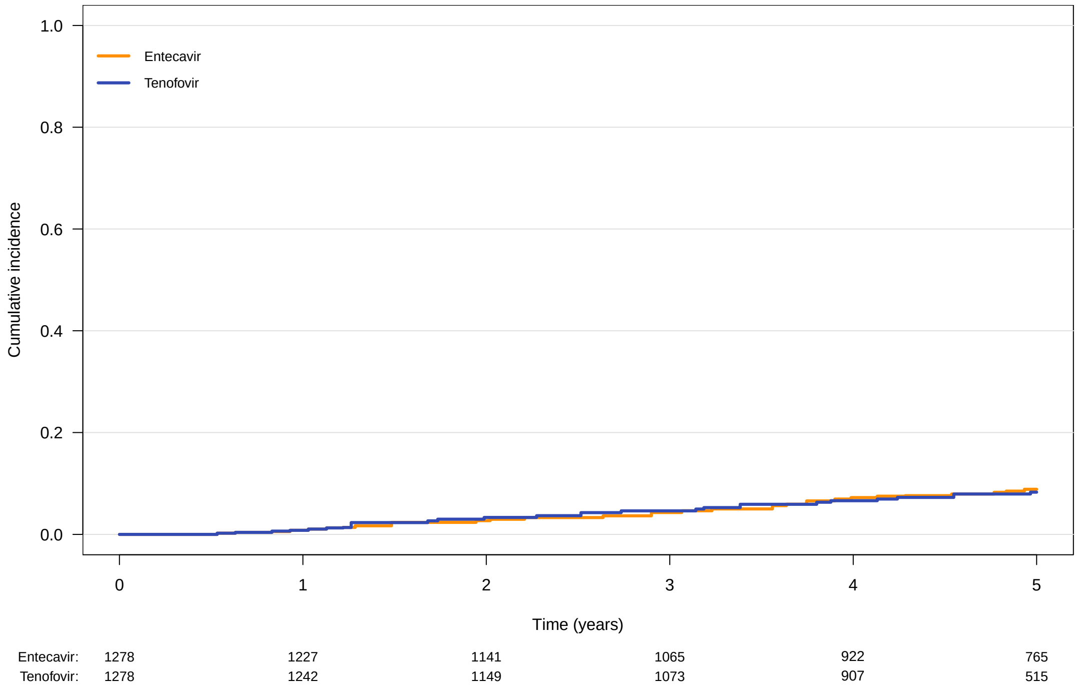

©2022 Tan DJH et al. *JAMA Network Open*.

Lee et al 2020

Original

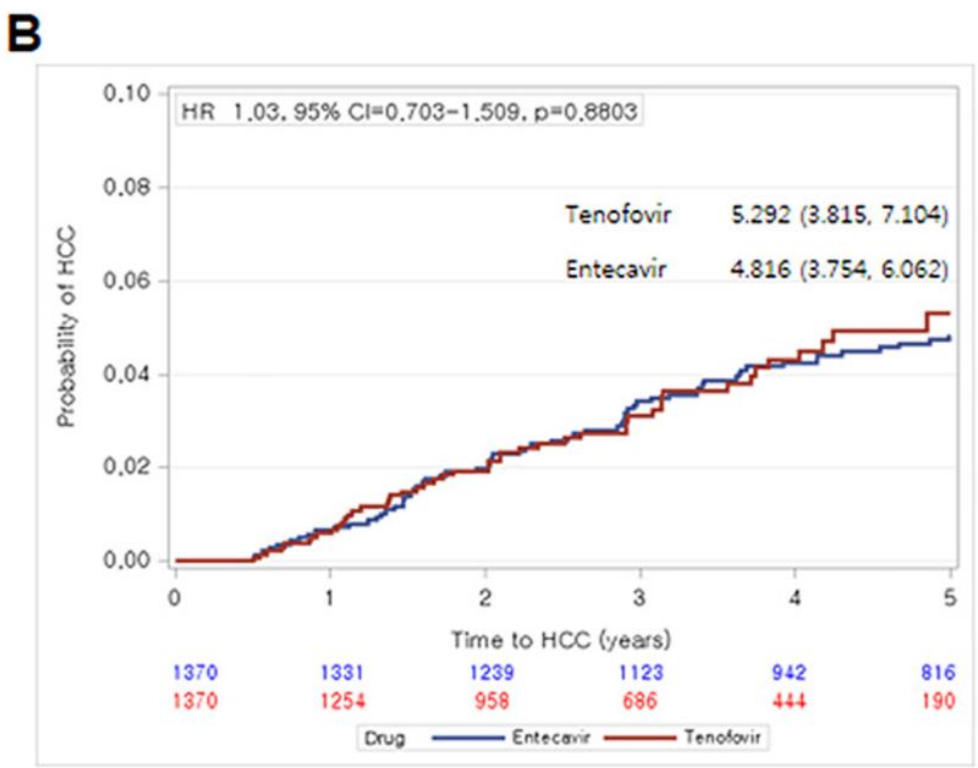

Reconstructed

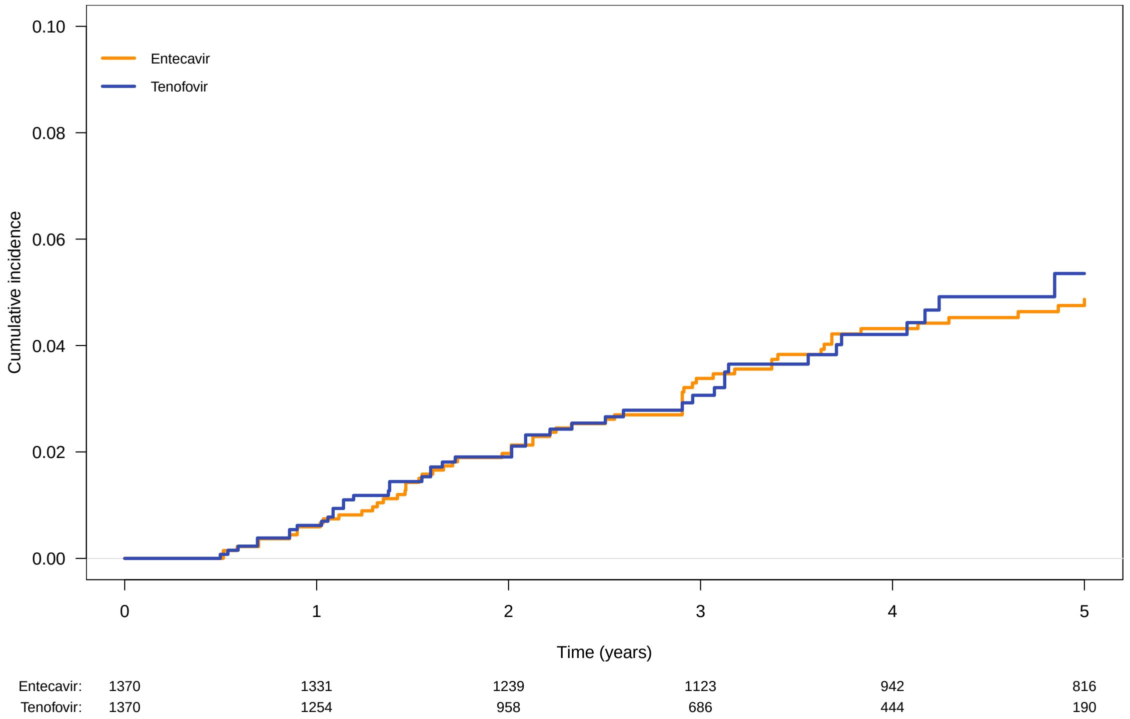

©2022 Tan DJH et al. *JAMA Network Open*.

Ha et al 2020

Original

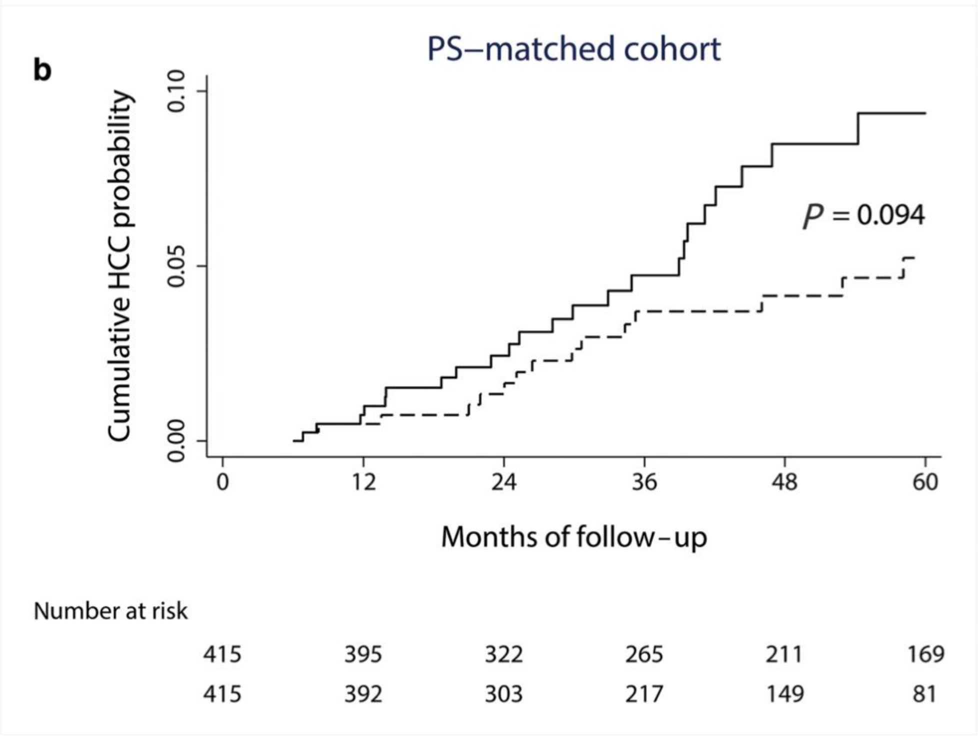

Reconstructed

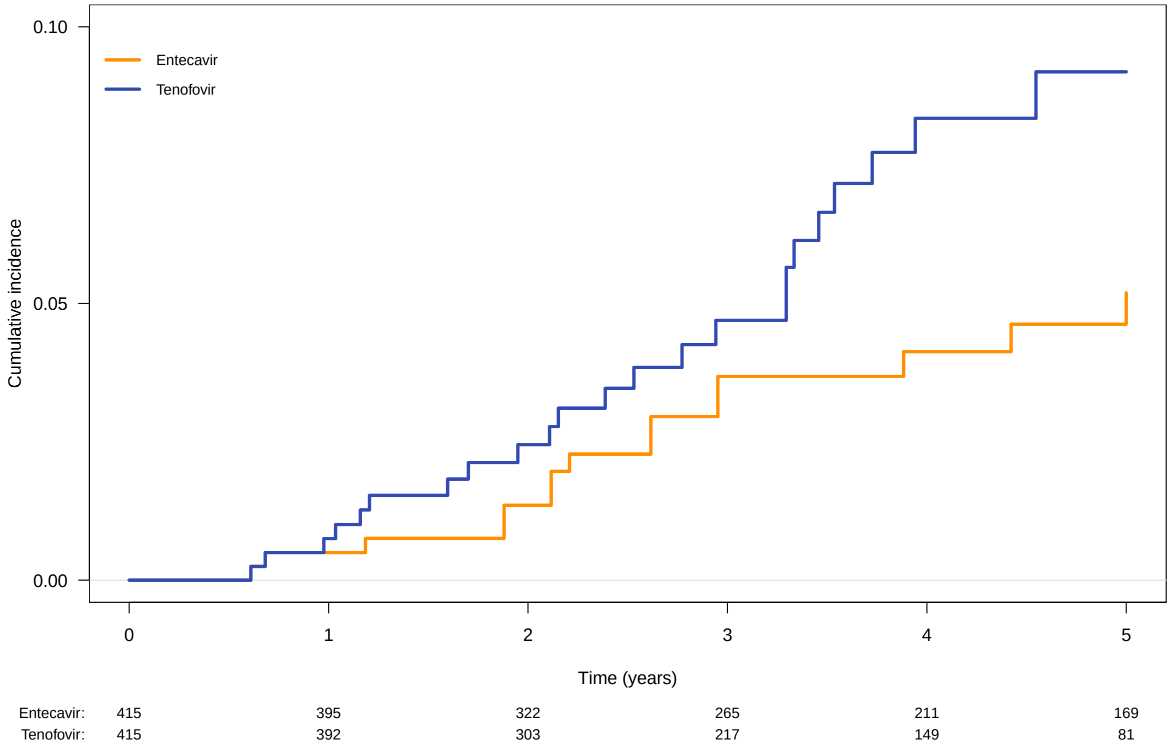

©2022 Tan DJH et al. *JAMA Network Open*.

Kim et al 2018

Original

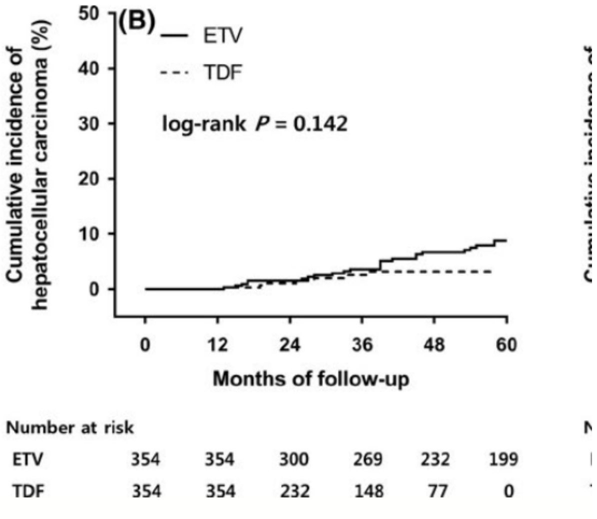

Reconstructed

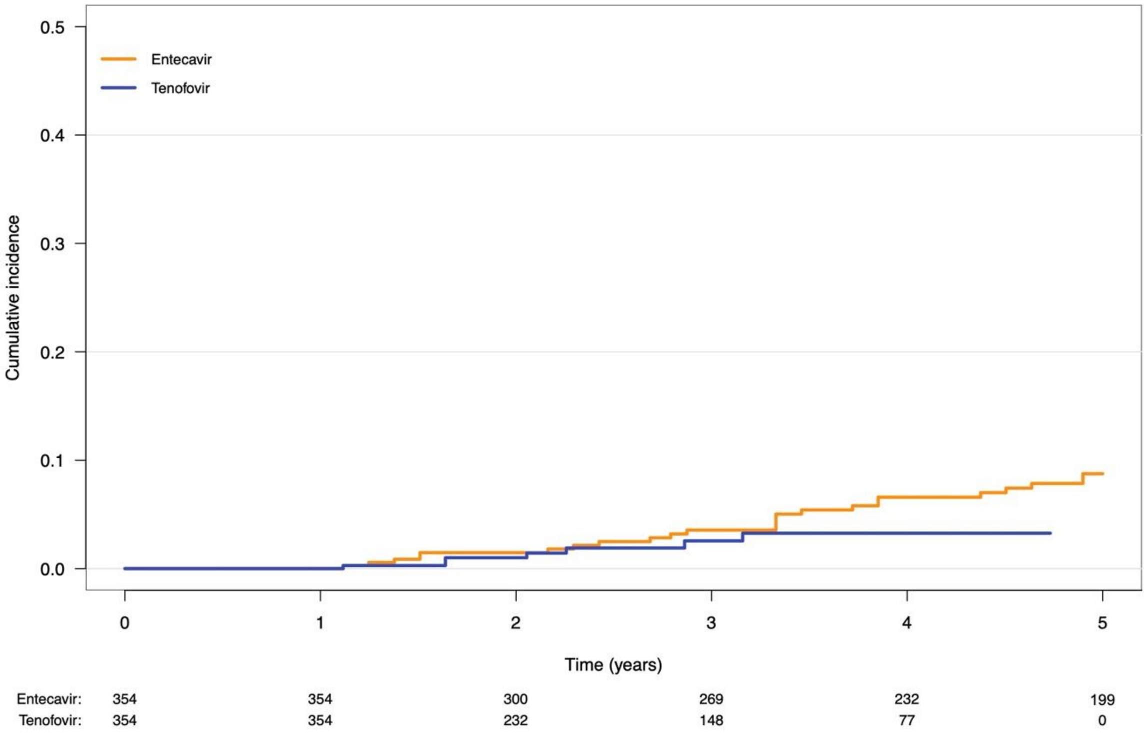

©2022 Tan DJH et al. *JAMA Network Open*.

Oh et al 2020

Original

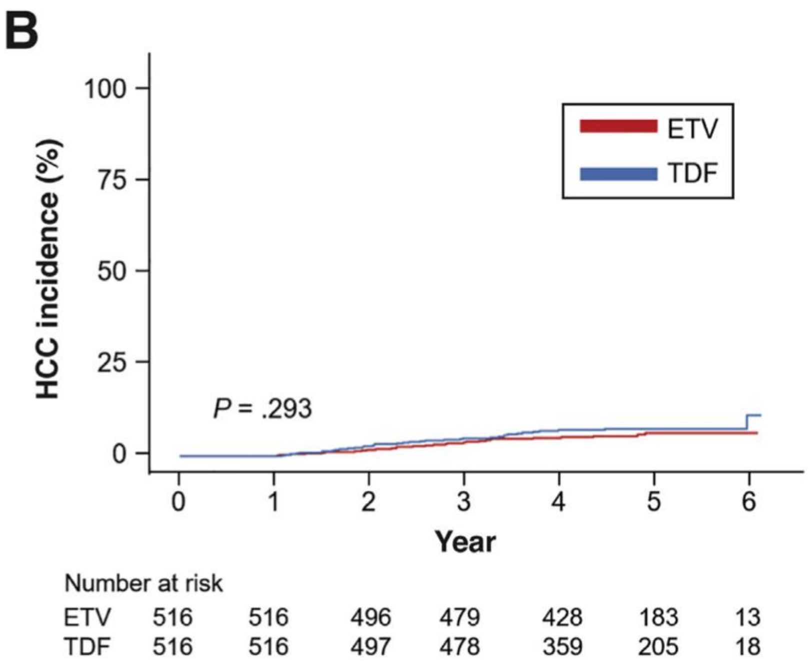

Reconstructed

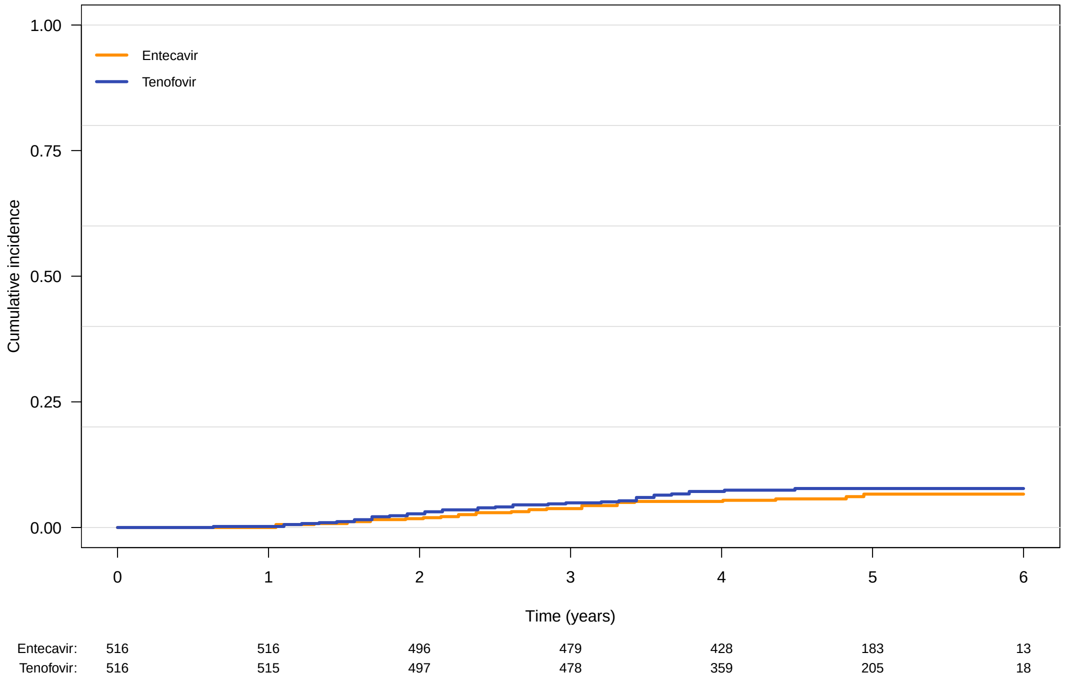

©2022 Tan DJH et al. *JAMA Network Open*.

Yip et al 2020

Original

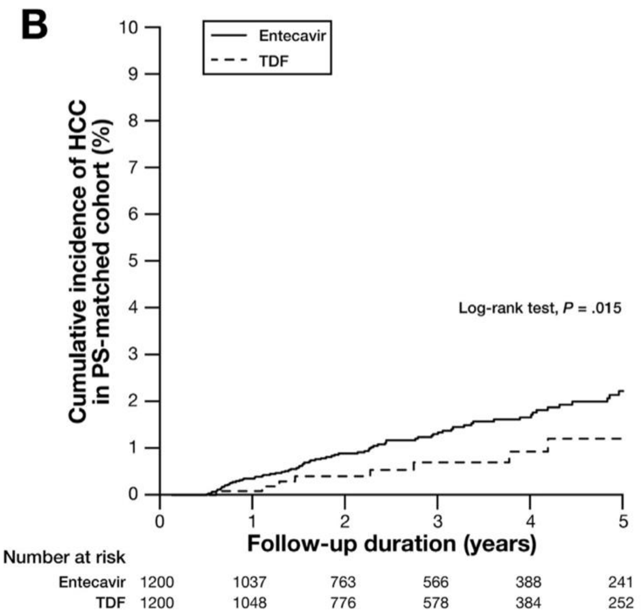

Reconstructed

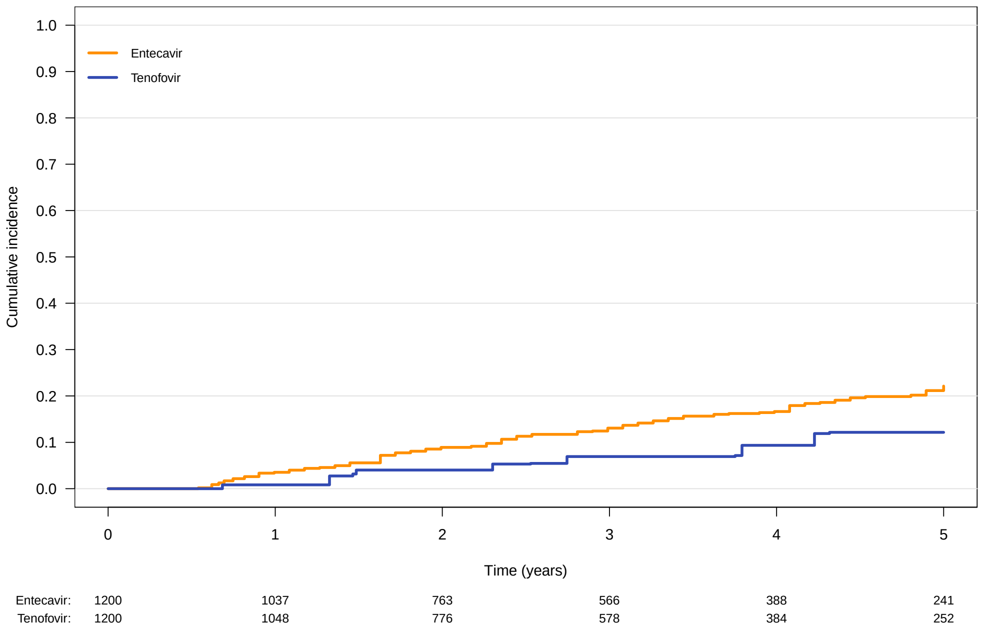

©2022 Tan DJH et al. *JAMA Network Open*.

Shin et al 2021

Original

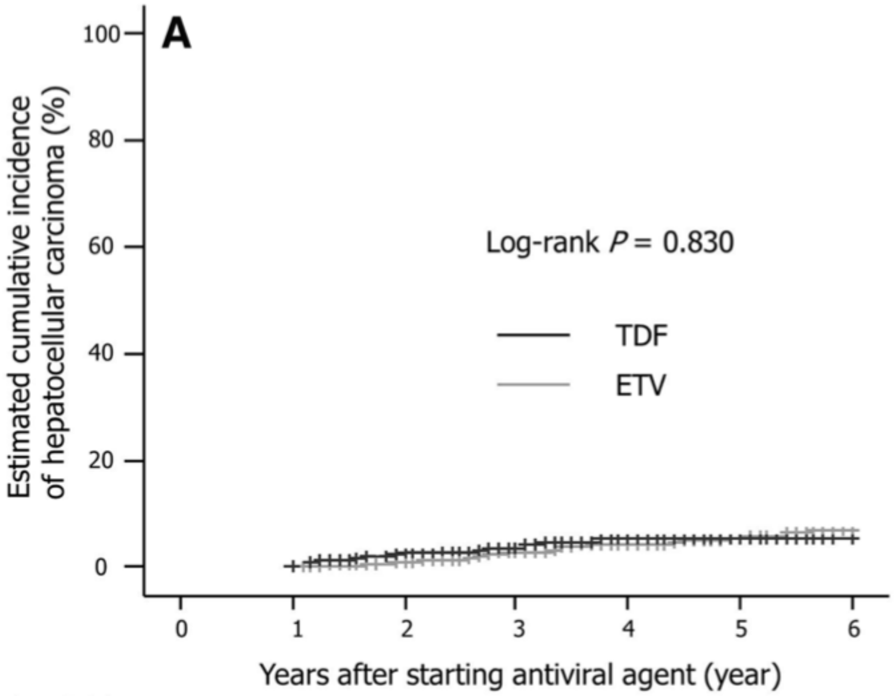

Reconstructed

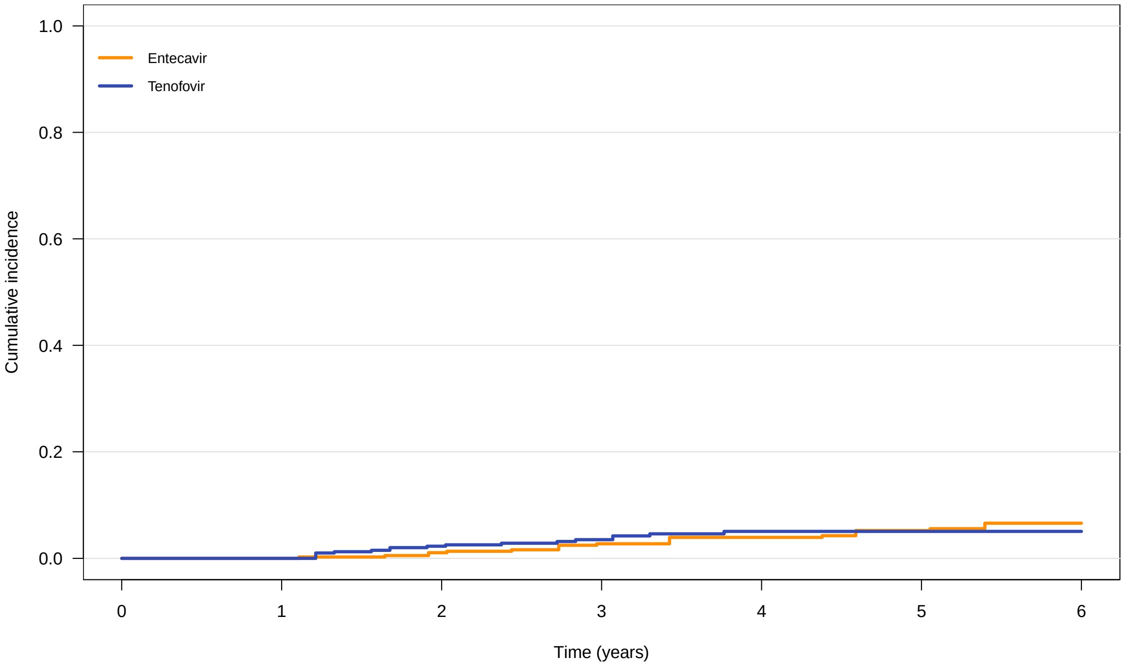

©2022 Tan DJH et al. *JAMA Network Open*.

Chen et al 2020

Original

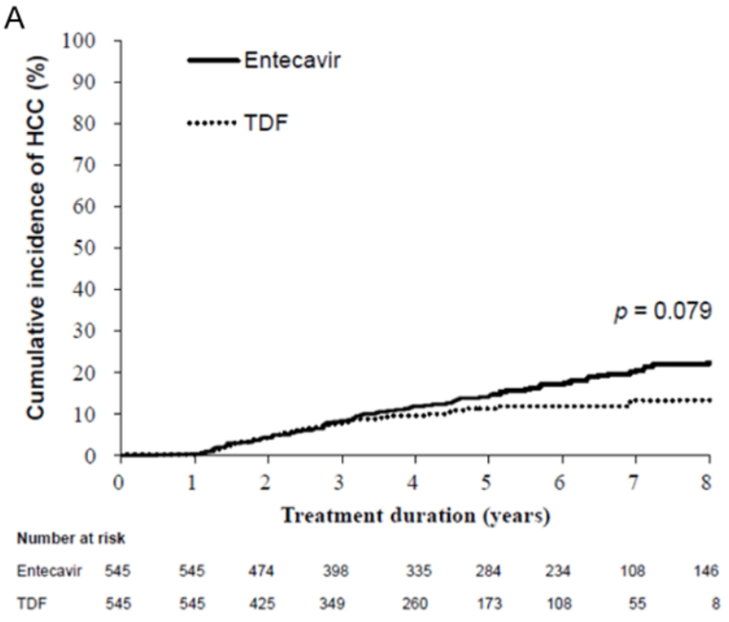

Reconstructed

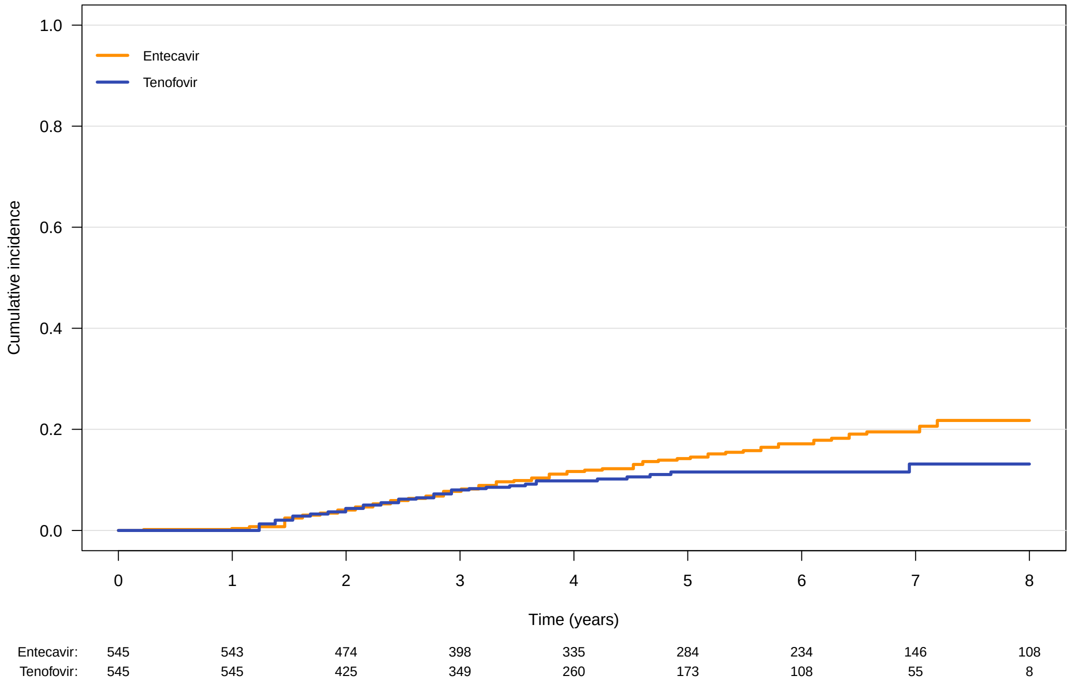

©2022 Tan DJH et al. *JAMA Network Open*.

Hu et al 2020

Original

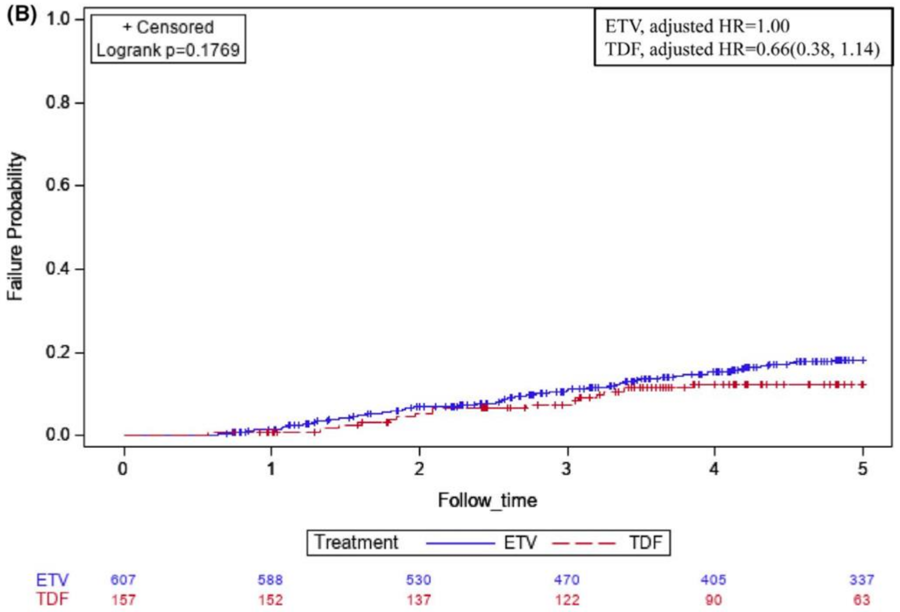

Reconstructed

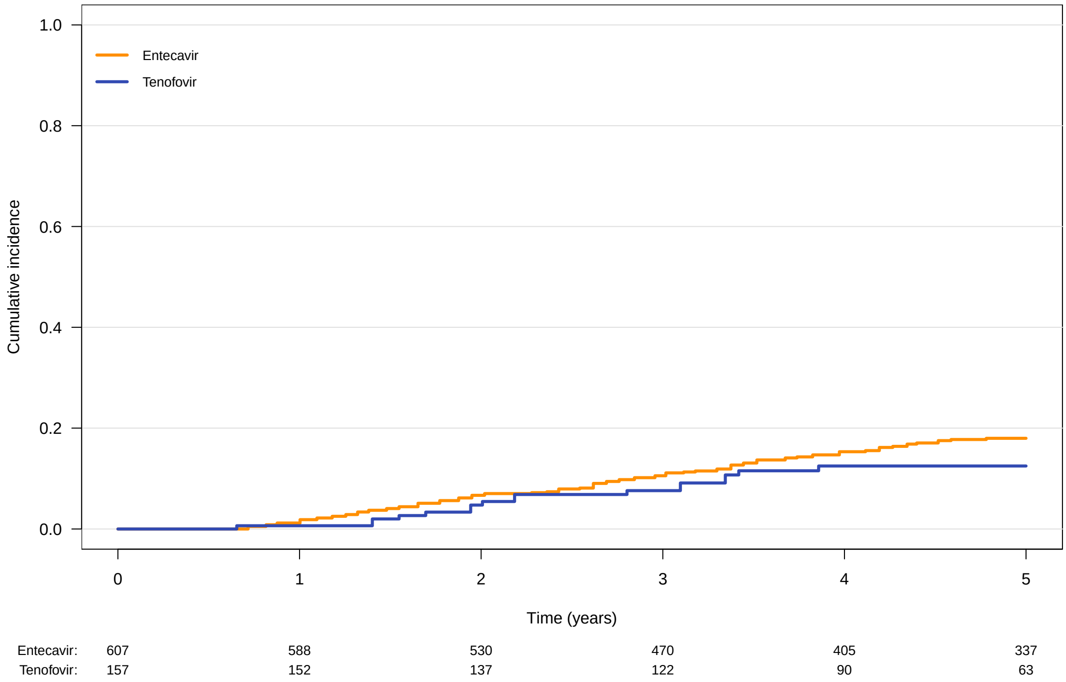

©2022 Tan DJH et al. *JAMA Network Open*.

Ha et al 2020

Original

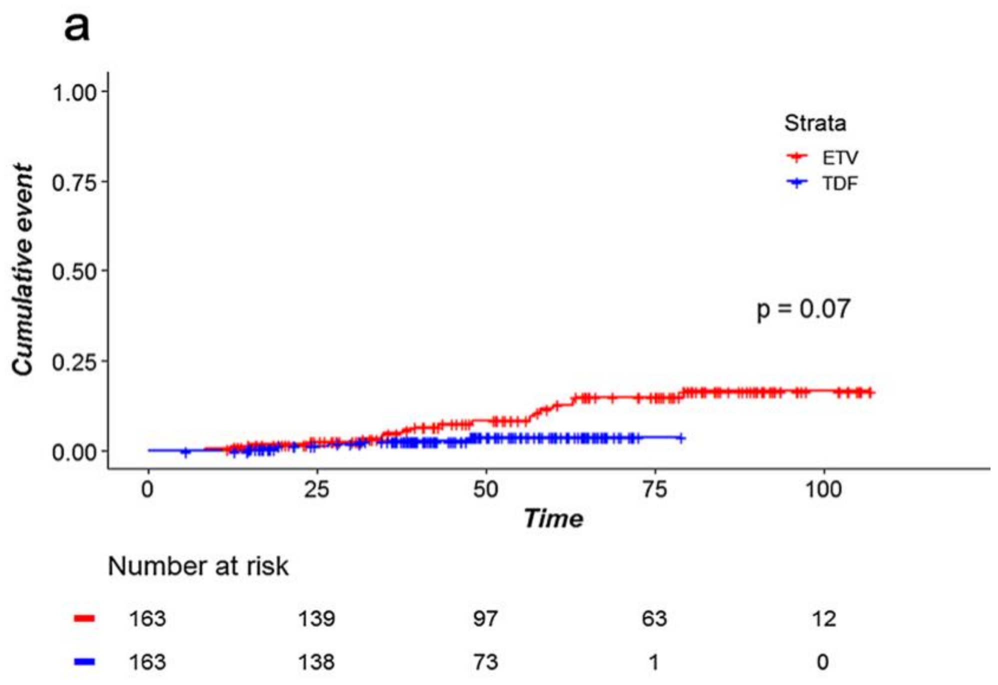

Reconstructed

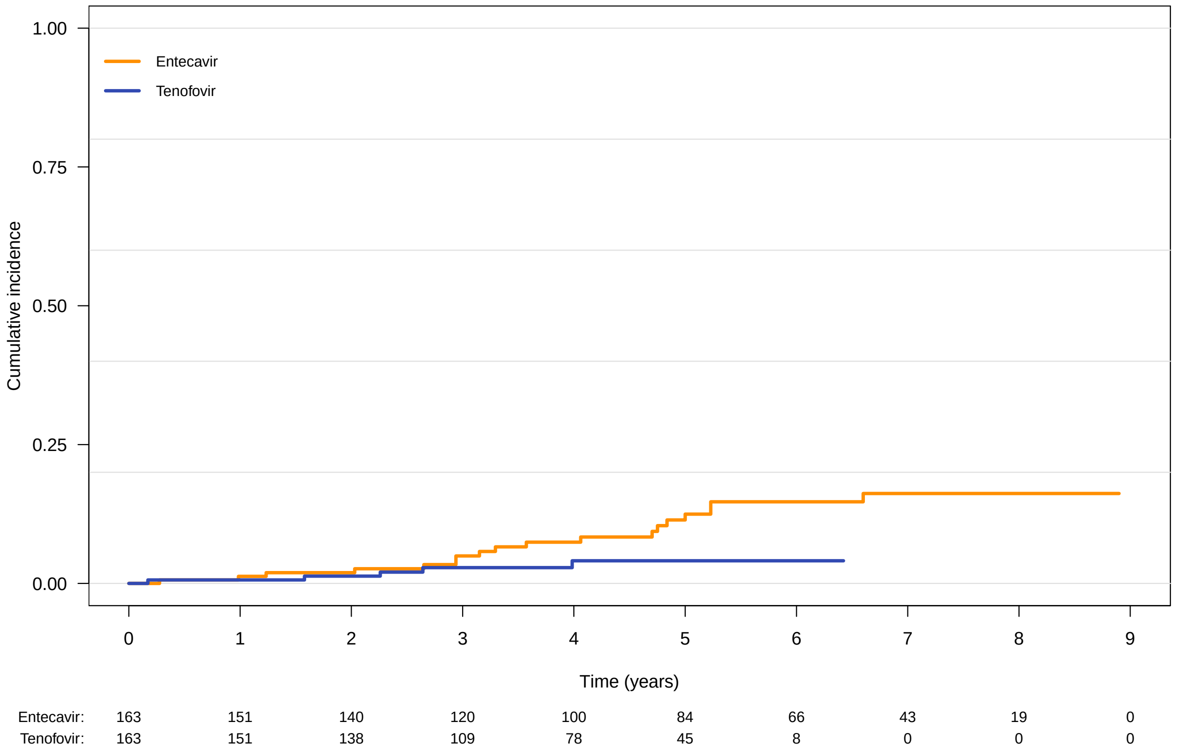

©2022 Tan DJH et al. *JAMA Network Open*.

Chang et al 2021

Original

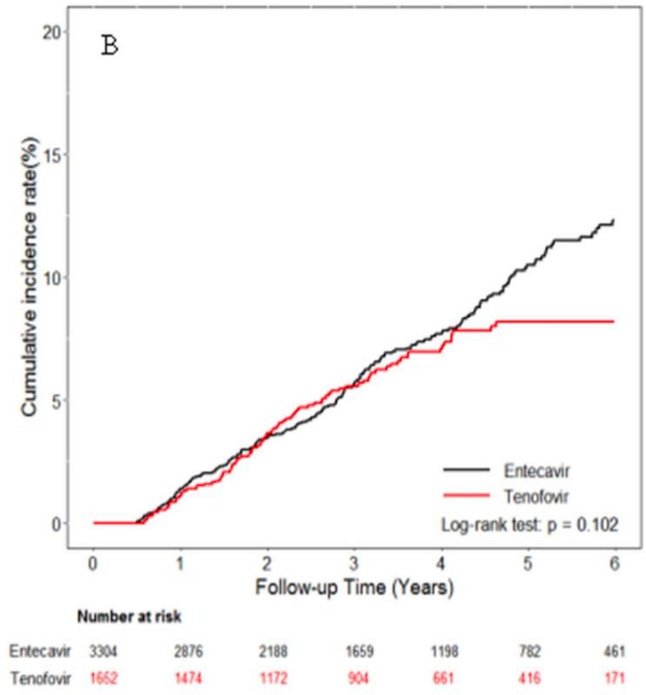

Reconstructed

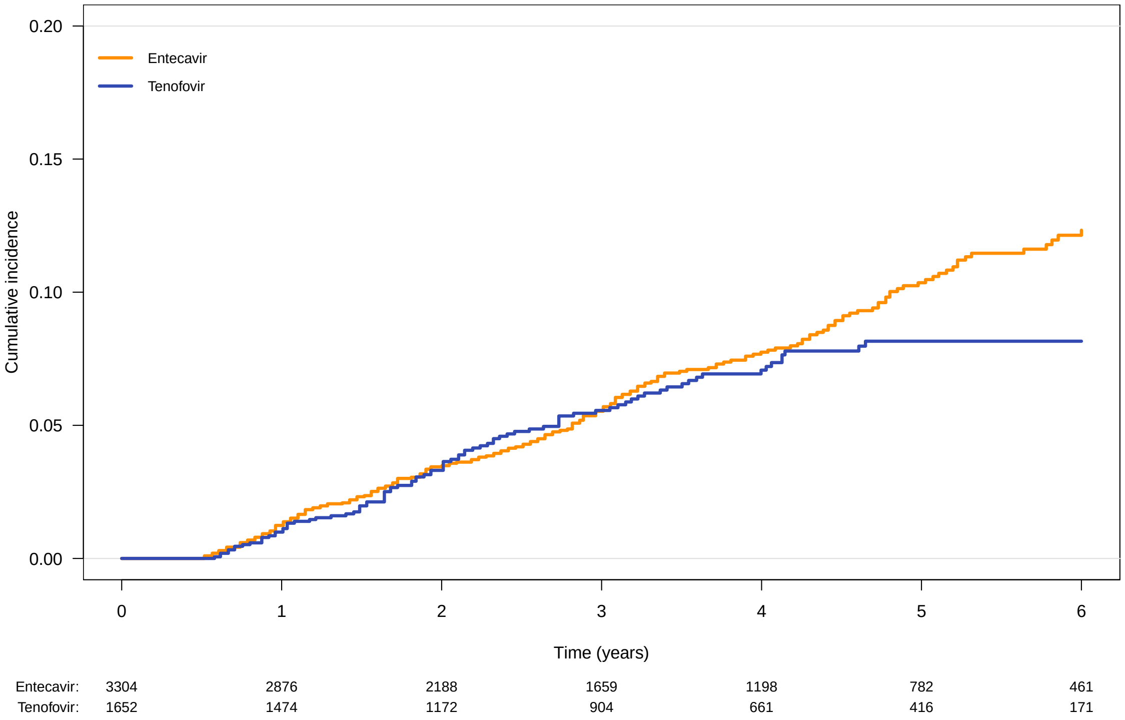

©2022 Tan DJH et al. *JAMA Network Open*.

Su et al 2021

Original

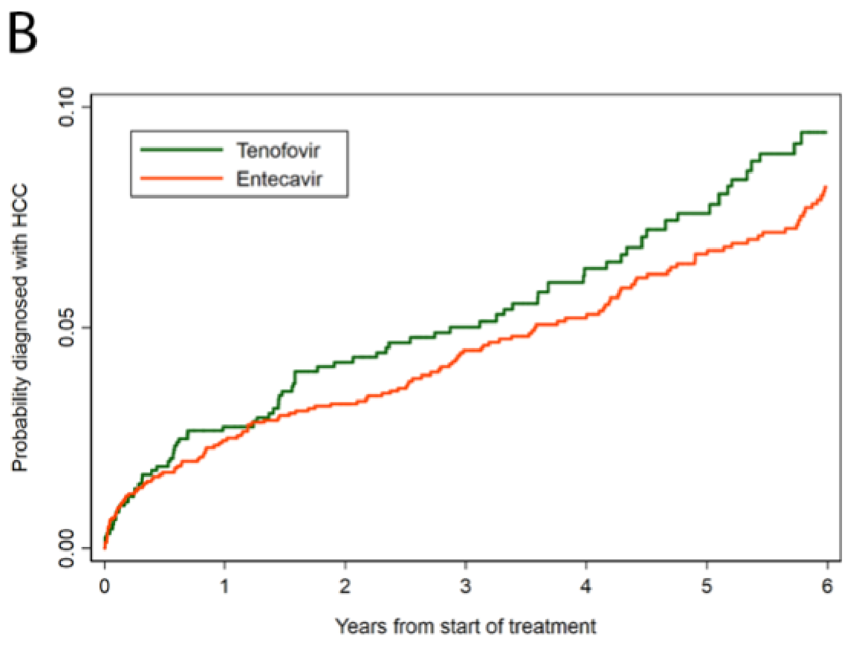

Reconstructed

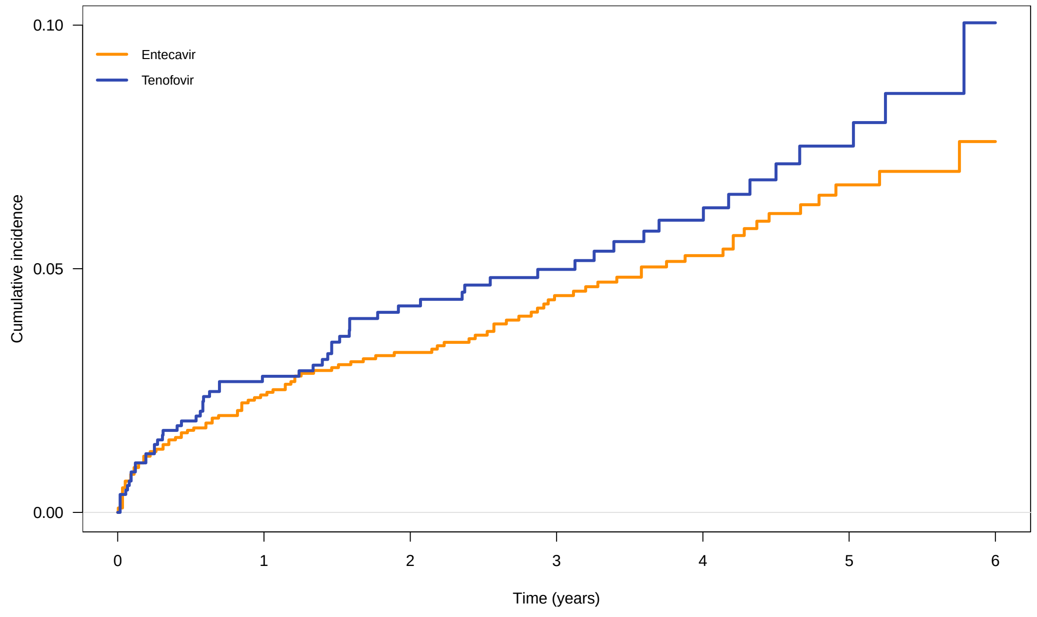

©2022 Tan DJH et al. *JAMA Network Open*.

eFigure 2. Assessment of Publication Bias

Funnel plot for analysis of the overall cohort

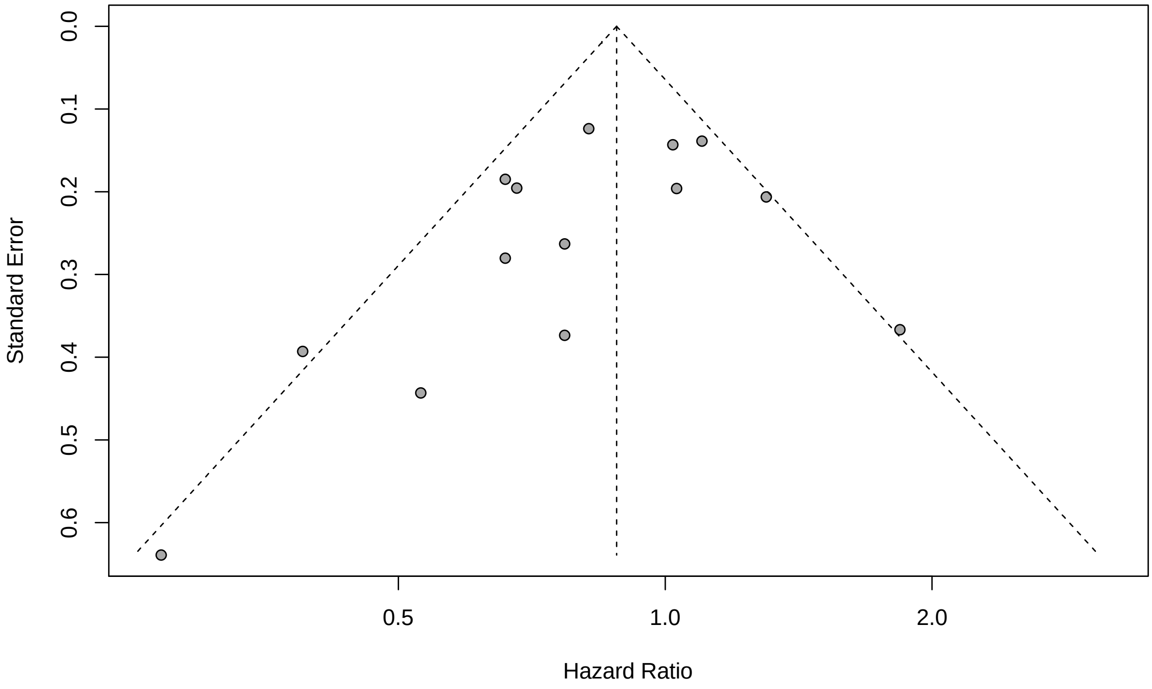

Funnel plot for sensitivity analysis of clinic cohort studies

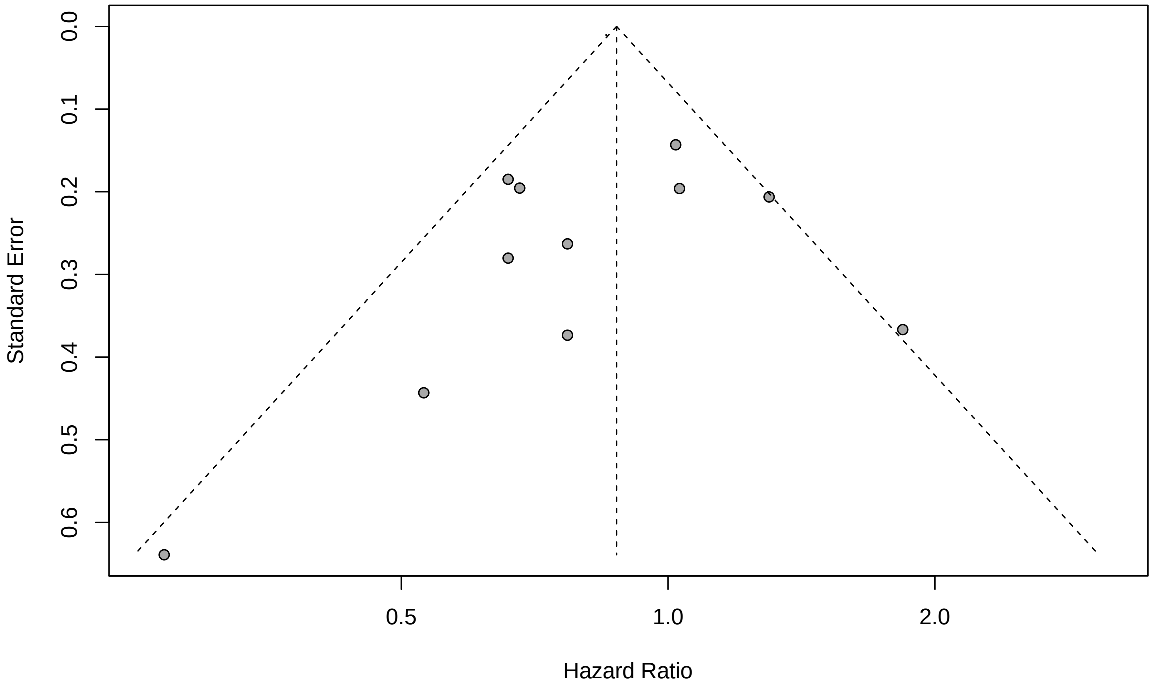

©2022 Tan DJH et al. *JAMA Network Open*.

Funnel plot for sensitivity analysis of studies involving only treatment-naïve patients

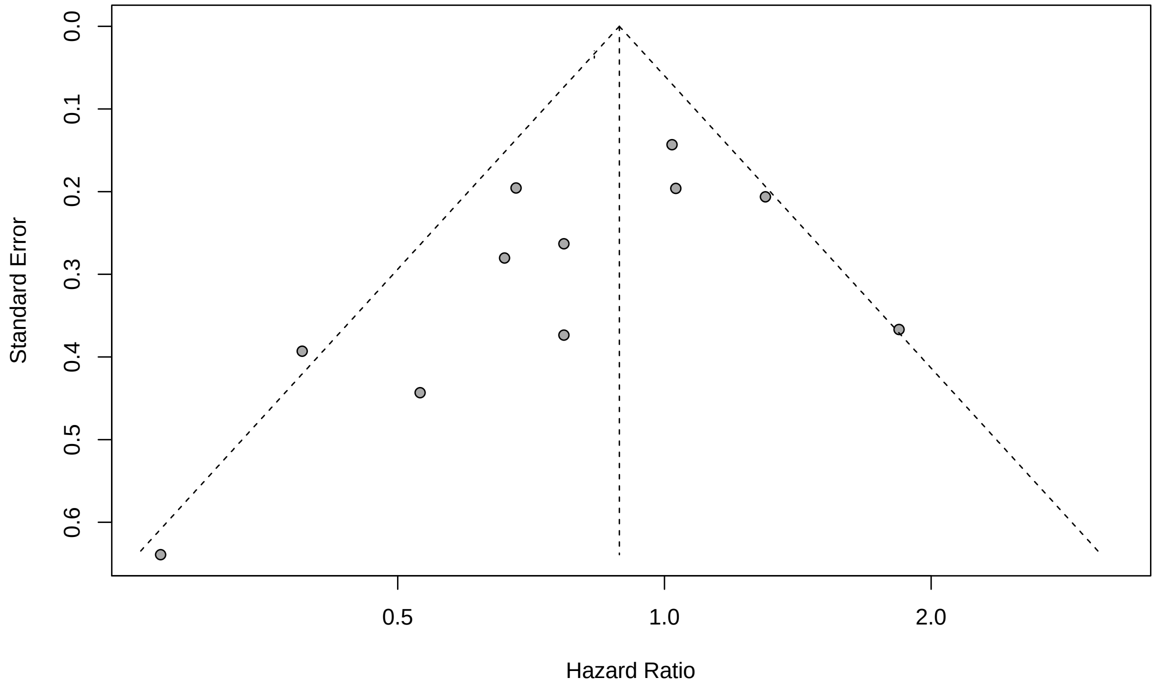

©2022 Tan DJH et al. *JAMA Network Open*.

eTable 2. Restricted Mean Survival Time (RMST) Analysis of HCC Incidence in Patients Treated With Tenofovir vs Entecavir

A. In overall cohort and patients with cirrhosis only

**Time/ years**	**RMST**		**RMST difference**	***p***	**RMST ratio**	***p***
	**Tenofovir**	**Entecavir**				
Overall cohort						
1	0.996 (0.995 - 0.997)	0.995 (0.994 - 0.996)	0.001 (0.000 to 0.003)	0.16	1.001 (1.000 to 1.003)	0.16
2	1.976 (1.973 - 1.979)	1.970 (1.967 - 1.973)	0.006 (0.001 to 0.010)	0.02	1.003 (1.001 to 1.005)	0.02
3	2.935 (2.929 - 2.942)	2.925 (2.919 - 2.932)	0.010 (0.001 to 0.019)	0.03	1.003 (1.000 to 1.007)	0.03
4	3.877 (3.866 - 3.888)	3.859 (3.847 - 3.868)	0.020 (0.005 to 0.035)	0.01	1.005 (1.001 to 1.009)	0.01
5	4.803 (4.786 - 4.819)	4.769 (4.755 - 4.784)	0.033 (0.011 to 0.055)	<0.001	1.007 (1.002 to 1.012)	<0.001
Cirrhosis only						
1	0.980 (0.975 - 0.985)	0.982 (0.977 - 0.986)	-0.002 (-0.008 to 0.005)	0.57	0.998 (0.992 to 1.005)	0.57
2	1.935 (1.924 - 1.947)	1.932 (1.922 - 1.943)	0.003 (-0.013 to 0.018)	0.74	1.001 (0.993 to 1.009)	0.74
3	2.855 (2.835 - 2.876)	2.845 (2.827 - 2.864)	0.010 (-0.018 to 0.038)	0.48	1.003 (0.994 to 1.013)	0.48
4	3.746 (3.714 - 3.777)	3.719 (3.691 - 3.748)	0.026 (-0.016 to 0.068)	0.23	1.007 (0.996 to 1.019)	0.23
5	4.615 (4.570 - 4.659)	4.565 (4.525 - 4.604)	0.050 (-0.010 to 0.109)	0.10	1.011 (0.988 to 1.024)	0.10

©2022 Tan DJH et al. *JAMA Network Open*.

B. Stratified by single-centre versus multicentre studies

Time/ years	RMST		RMST difference	*p*	RMST ratio	*p*
	Tenofovir	Entecavir				
Single centre studies						
1	0.996 (0.994- 0.998)	0.996 (0.994- 0.998)	0.000 (-0.003 to 0.002)	0.87	1.000 (0.997 to 1.002)	0.87
2	1.981 (1.976 - 1.986)	1.977 (1.972 - 1.982)	0.004 (-0.003 to 0.011)	0.26	1.002 (0.999 to 1.006)	0.26
3	2.948 (2.938 - 2.958)	2.937 (2.927 - 2.947)	0.011 (-0.003 to 0.026)	0.12	1.004 (0.999 to 1.009)	0.12
4	3.898 (3.881 - 3.915)	3.874 (3.858 - 3.891)	0.023 (0.000 to 0.047	0.05	1.006 (1.000 to 1.012)	0.05
5	4.836 (4.810 - 4.861)	4.794 (4.770 - 4.818)	0.042 (0.006 to 0.077)	0.02	1.009 (1.001 to 1.016)	0.02
Multicentre studies						
1	0.996 (0.994 - 0.997)	0.994 (0.993 - 0.995)	0.002 (0.000 to 0.003)	0.07	1.002 (1.000 to 1.003)	0.07
2	1.973 (1.969 - 1.977)	1.967 (1.963 - 1.971)	0.006 (0.000 to 0.012)	0.05	1.003 (1.000 to 1.006)	0.05
3	2.928 (2.920 - 2.937)	2.920 (2.912 - 2.982)	0.008 (-0.004 to 0.020)	0.18	1.003 (0.999 to 1.007)	0.18
4	3.866 (3.852 - 3.880)	3.849 (3.837 - 3.862)	0.017 (-0.002 to 0.036)	0.09	1.004 (0.999 to 1.009)	0.09
5	4.785 (4.765 - 4.806)	4.758 (4.739 - 4.776)	0.028 (0.000 to 0.056)	0.05	1.006 (1.000 to 1.012)	0.05

©2022 Tan DJH et al. *JAMA Network Open*.

C. In studies with treatment naïve patients only

Time/ years	RMST		RMST difference	*p*	RMST ratio	*p*
	Tenofovir	Entecavir				
Clinical cohort						
1	0.997 (0.996 - 0.998)	0.996 (0.995 - 0.997)	0.001 (-0.001 to 0.002)	0.29	1.001 (0.999 to 1.002)	0.29
2	1.980 (1.976 - 1.983)	1.973 (1.969 - 1.977)	0.007 (0.002 to 0.012)	0.01	1.003 (1.001 to 1.006)	0.01
3	2.943 (2.936 - 2.950)	2.927 (2.919 - 2.935)	0.016 (0.005 to 0.027)	0.004	1.005 (1.002 to 1.009)	0.004
4	3.889 (3.877 - 3.901)	3.858 (3.845 - 3.871)	0.031 (0.013 to 0.049)	<0.001	1.008 (1.003 to 1.013)	<0.001
5	4.817 (4.799 - 4.836)	4.769 (4.750 - 4.788)	0.049 (0.022 to 0.075)	<0.001	1.010 (1.005 to 1.016)	<0.001

©2022 Tan DJH et al. *JAMA Network Open*.

eFigure 3. Cumulative Incidence of HCC in Patients Receiving Tenofovir vs Entecavir in Patients With Cirrhosis Only

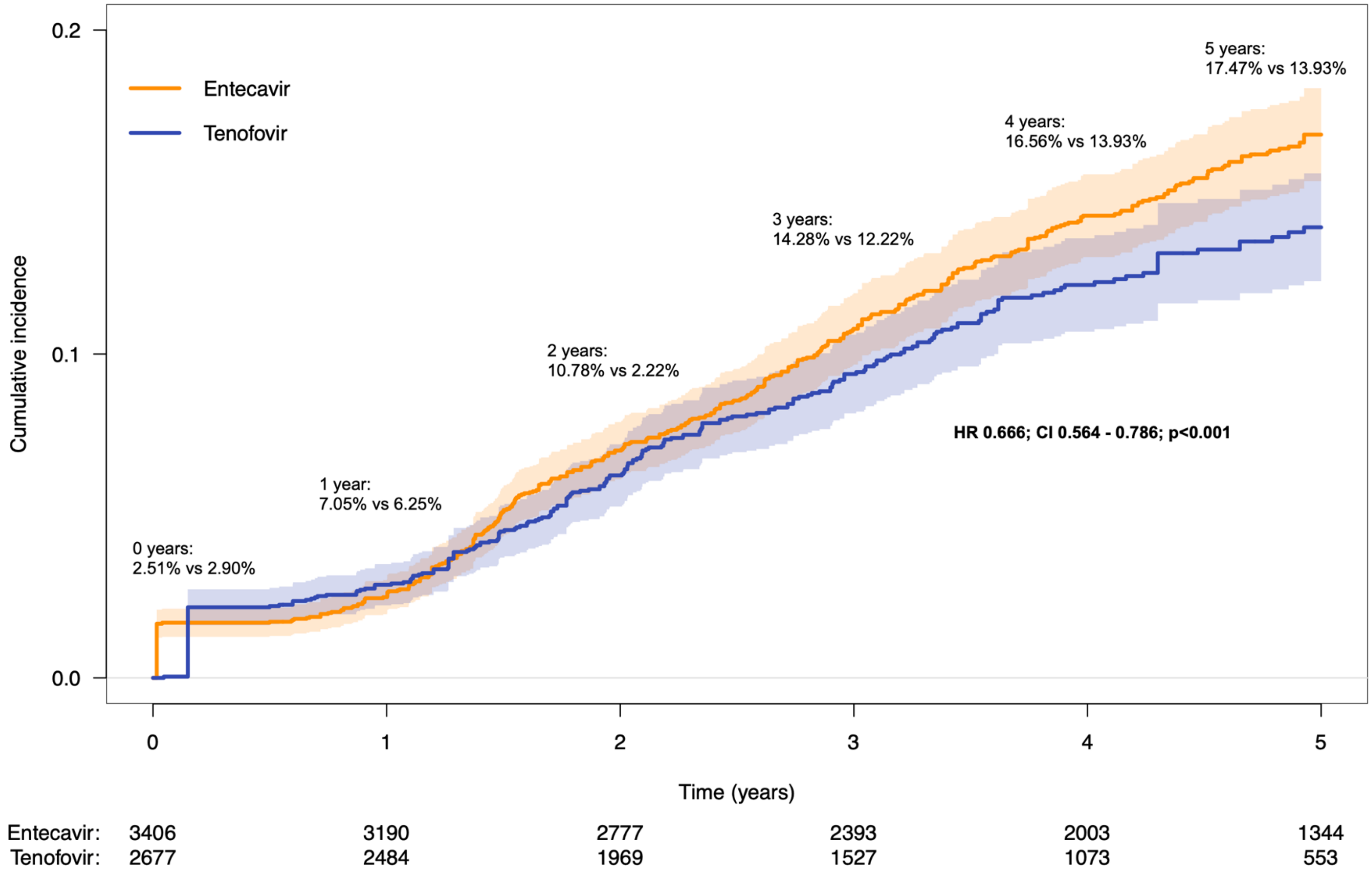

©2022 Tan DJH et al. *JAMA Network Open*.

eFigure 4. Cumulative Incidence of HCC in Patients Receiving Tenofovir vs Entecavir in (A) Single Centre Studies and in (B) Multicentre Studies

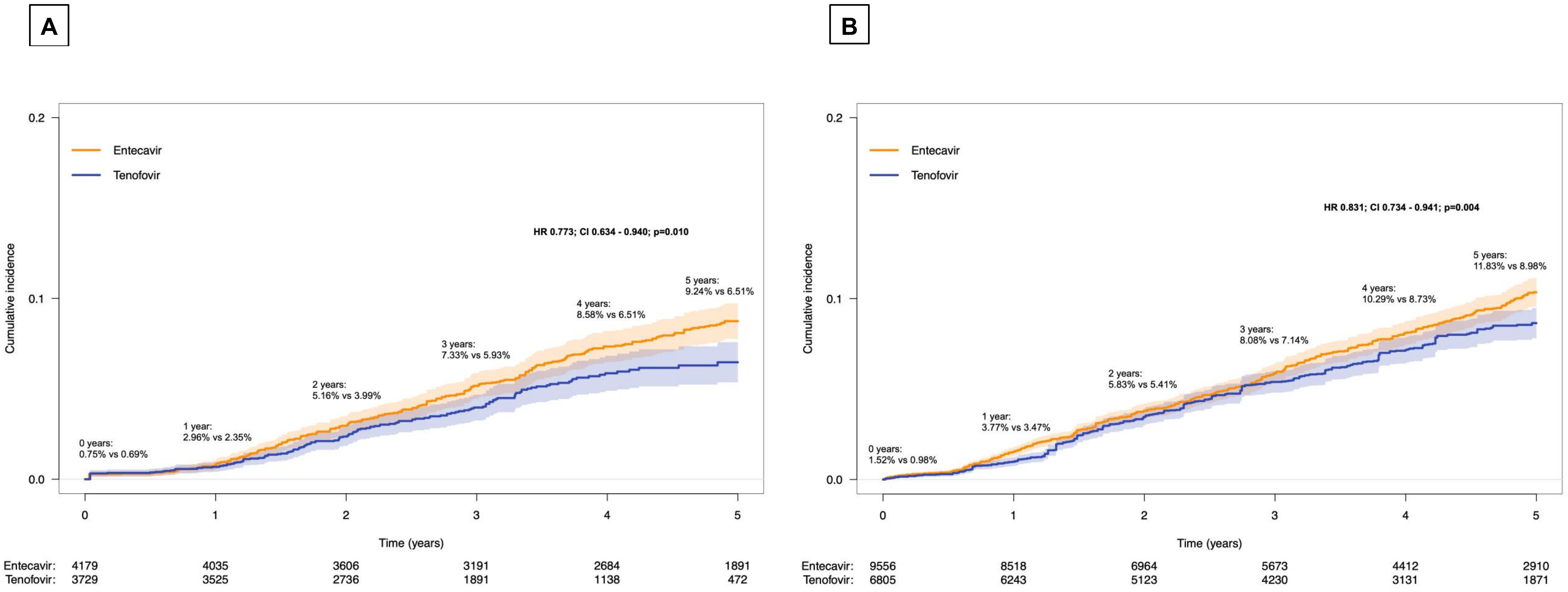

©2022 Tan DJH et al. *JAMA Network Open*.

eFigure 5. Cumulative Incidence of HCC in Patients Receiving Tenofovir vs Entecavir in Studies Involving Treatment-Naïve Patients Only

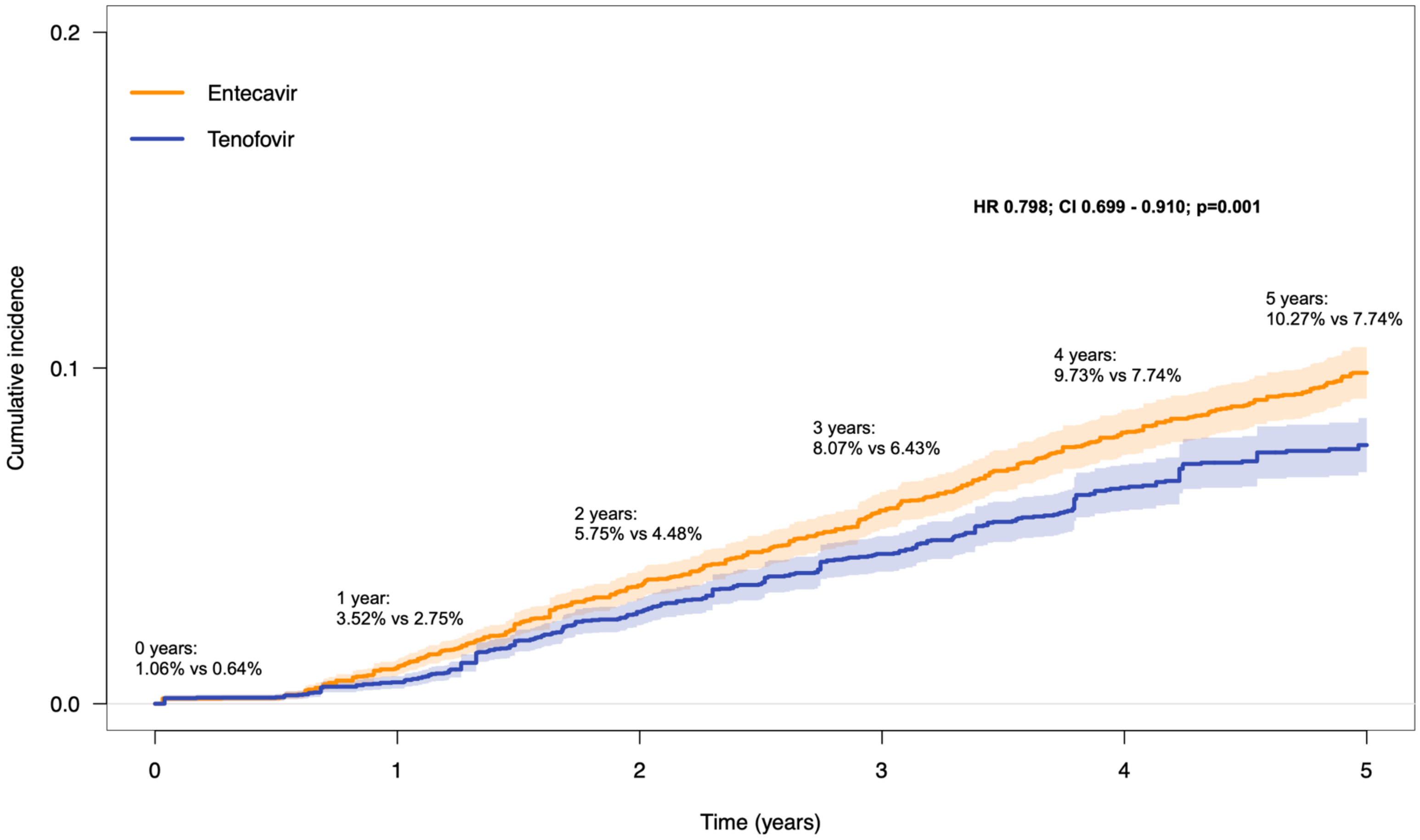

Entecavir:	7693	7335	6462	5702	4811	3327
Tenofovir:	7243	6850	5547	4323	2979	1560

©2022 Tan DJH et al. *JAMA Network Open*.

References.

1. Hsu YC, Wong GLH, Chen CH, et al. Tenofovir Versus Entecavir for Hepatocellular Carcinoma Prevention in an International Consortium of Chronic Hepatitis B. The American journal of gastroenterology. 2020;115(2):271-280.
2. Choi J, Kim HJ, Lee J, Cho S, Ko MJ, Lim YS. Risk of Hepatocellular Carcinoma in Patients Treated with Entecavir vs Tenofovir for Chronic Hepatitis B: A Korean Nationwide Cohort Study. JAMA Oncology. 2019;5(1):30-36.
3. Kim SU, Seo YS, Lee HA, et al. A multicenter study of entecavir vs. tenofovir on prognosis of treatment-naïve chronic hepatitis B in South Korea. Journal of Hepatology. 2019;71(3):456-464.
4. Lee SW, Kwon JH, Lee HL, et al. Comparison of tenofovir and entecavir on the risk of hepatocellular carcinoma and mortality in treatment-naïve patients with chronic hepatitis B in Korea: A large-scale, propensity score analysis. Gut. 2020;69(7):1301-1308.
5. Ha I, Chung JW, Jang ES, Jeong SH, Kim JW. Comparison of the on-treatment risks for hepatocellular carcinoma between entecavir and tenofovir: A propensity score matching analysis. Journal of Gastroenterology and Hepatology (Australia). 2020;35(10):1774-1781.
6. Kim BG, Park NH, Lee SB, et al. Mortality, liver transplantation and hepatic complications in patients with treatment-naïve chronic hepatitis B treated with entecavir vs tenofovir. Journal of Viral Hepatitis. 2018;25(12):1565-1575.
7. Oh H, Yoon EL, Jun DW, et al. No Difference in Incidence of Hepatocellular Carcinoma in Patients With Chronic Hepatitis B Virus Infection Treated With Entecavir vs Tenofovir. Clinical Gastroenterology and Hepatology. 2020;18(12):2793-2802.e2796.
8. Yip TCF, Wong VWS, Chan HLY, Tse YK, Lui GCY, Wong GLH. Tenofovir Is Associated With Lower Risk of Hepatocellular Carcinoma Than Entecavir in Patients With Chronic HBV Infection in China. Gastroenterology. 2020;158(1):215-225.e216.
9. Shin JW, Jeong J, Jung SW, et al. Comparable Incidence of Hepatocellular Carcinoma in Chronic Hepatitis B Patients Treated with Entecavir or Tenofovir. Digestive Diseases and Sciences. 2021;66(5):1739-1750.
10. Chen CH, Chen CY, Wang JH, et al. Comparison of incidence of hepatocellular carcinoma between chronic hepatitis B patients with cirrhosis treated with entecavir or tenofovir in Taiwan - A retrospective study. American Journal of Cancer Research. 2020;10(11):3882-3895.
11. Hu TH, Yueh-Hsia Chiu S, Tseng PL, et al. Five-year comparative risk of hepatocellular carcinoma development under entecavir or tenofovir treatment-naïve patients with chronic hepatitis B-related compensated cirrhosis in Taiwan. Alimentary Pharmacology and Therapeutics. 2020;52(11-12):1695-1706.
12. Ha Y, Chon YE, Kim MN, Lee JH, Hwang SG. Hepatocellular carcinoma and death and transplantation in chronic hepatitis B treated with entecavir or tenofovir disoproxil fumarate. Scientific reports. 2020;10(1):13537.
13. Chang TS, Yang YH, Chen WM, et al. Long-term risk of primary liver cancers in entecavir versus tenofovir treatment for chronic hepatitis B. Scientific reports. 2021;11(1):1365.

©2022 Tan DJH et al. *JAMA Network Open*.

14. Su F, Berry K, Ioannou GN. No difference in hepatocellular carcinoma risk between chronic hepatitis B patients treated with entecavir versus tenofovir. Gut. 2021;70(2):370-378.

©2022 Tan DJH et al. *JAMA Network Open*.
